# Network pharmacology–driven identification of PTP1β and rational design of simufilam-based allosteric inhibitors for neuroprotection in Alzheimer’s disease

**DOI:** 10.1007/s13205-026-05086-2

**Published:** 2026-10-15

**Authors:** Vishnu Malakar, Rajeshkumar Raman, Antony Justin, Dhanabal SP, Dhritiman Roy, Chandi C. Malakar, Dipen Purohit, Pratap Chand Mali

**Affiliations:** 1 Department of Pharmacognosy, JSS College of Pharmacy, JSS Academy of Higher Education and Research, The Nilgiris, Ooty, 643001 Tamil Nadu India; 2 Department of Pharmaceutical Biotechnology, JSS College of Pharmacy, JSS Academy of Higher Education and Research, Nilgiris, Ooty, Tamil Nadu India; 3 Centre for Neuropharmacology and Experimental Neuroscience, Department of Pharmacology, JSS College of Pharmacy, JSS Academy of Higher Education and Research, The Nilgiris, Ooty, 643001 Tamil Nadu India; 4 Department of Pharmaceutical Sciences, Faculty of Science and Engineering, Dibrugarh University, Dibrugarh, 786004 Assam India; 5 Department of Chemistry, National Institute of Technology Manipur, Imphal, 795004 Manipur India; 6 Analytical Research & Quality Control Navinta LLC, 1499 Lower Ferry Road, Ewing, NJ 08618 USA; 7 Reproductive Biomedicine &amp, Natural Product Laboratory, Department of Zoology, University of Rajasthan, Jaipur, 302004 India

**Keywords:** Simufilam derivatives, PTP1B inhibition, MD simulation 1000ns, MM/PBSA, DFT calculations

## Abstract

Alzheimer’s disease (AD) is a progressive neurodegenerative disorder that predominantly affects elderly people and is characterized by cognitive decline and dementia. Protein tyrosine phosphatase-1B (PTP1B) is known to modulate pro-inflammatory mediators, and its overexpression in the brain has been implicated in AD-associated neuroinflammation. This study explores the mechanism of A20, A26, and co-crystal-designed compound targeting non-competitive allosteric PTP1B enzyme inhibition for AD therapy using network pharmacology and computational studies. Protein-protein interaction was designed for 128 active targets by STRING database and Cytoscape software. Further, active target-associated pathways analysed using ClueGO plugin Cytoscape software and the Reactome online public domain server. The non-competitive allosteric PTP1B inhibition through the design of Simufilam derivatives, with particular emphasis on A20, which is predicted to bind at the non-competitive allosteric site of the PTP1B protein (PDB ID: 1T49), involves α3, α6, and α7 helices that influence the WPD loop orientation. The proposed non-competitive allosteric binding mode is based on computational evidence and for that further experimental validation would be required to conclusively confirm binding of the designed compounds within this regulatory region. Using in-silico approaches, including molecular docking, identified A20 with the most favourable predicted binding energy score (−10.8 kcal/mol) compared to other derivatives. Molecular dynamics simulations further indicated structural stability of the PTP1B-A20, 26, and co-crystal complex, with consistent RMSD, RMSF, H-bond, Rg, and SASA. Principal component analysis suggested that A20 restricts conformational flexibility; hence MM-PBSA analysis displayed its stable binding free energies. Density functional theory (DFT) calculation characterized the polarity, stability, and reactivity of A20 studies supported its favourable electronic configuration. Overall, A20 displayed superior calculated binding energies, trajectory stability, and predicted drug-likeness, positioning it as a promising lead candidate for future empirical validation in AD research.

**Supplementary Information:**

The online version contains supplementary material available at https://doi.org/10.1007/s13205-026-05086-2.

## Introduction

Alzheimer’s disease (AD) is an irreversible neurodegenerative disorder characterised by the accumulation of amyloid beta (Aβ) aggregates, insoluble tau protein-based neurofibrillary tangles (NFTs), and associated pathology in the hippocampal regions of the brain (Reddy et al. 2019). The accumulation of aggregated proteins is neurotoxic, leading to AD symptoms including cognitive impairment, dementia, poor judgment, personality changes, memory loss, behavioural changes, and reduced ability to perform self-care (Zhao et al. 2022). Despite the increasing number of AD patients, the condition remains a significant burden on individuals and society. Therefore, there is an urgent need to better understand the pathophysiology of AD and to develop more effective therapeutic agents (Sharallah et al. 2026). Currently, several FDA-approved drugs are available for the treatment of Alzheimer’s disease, including symptomatic therapies and disease-modifying agents (Malakar et al. 2025b). These include acetylcholinesterase inhibitors (AChEIs) such as donepezil (Agrawal et al. 2018), galantamine (Zhang and Gordon 2018), and rivastigmine (Liu et al. 2019); the N-methyl-D-aspartate (NMDAR) antagonist memantine (Lu and Nasrallah 2018), displaying potential therapeutic changes in cognitive decline and daily routine by reinstituting neurotransmitter alteration. amyloid-beta (Aβ) inhibitors such as aducanumab (Brockmann et al. 2023); and lecanemab (Brockmann et al. 2023); and GV-971, a gut microbiota modulator approved only in China (Syed 2020). Pharmacotherapies for AD approved drugs mainly divided into two classes: traditional symptomatic therapy and newer disease-modifying treatments (DMTs). Symptomatic therapy anticipates with acetylcholinesterase inhibitors (donepezil, rivastigmine, galantamine) and the NMDA receptor antagonist memantine, which temporarily regulate cognitive activity and daily routines by reinstating neurotransmitter levels (Birks and Harvey 2006). Recently, anti-Aβ mAbs including lecanemab have gained approval, representing that targeted amyloid plaque clearance can halt clinical deficits in early-stage AD (van Dyck et al. 2023). However, practical clinical limitations endure across both classes. Symptomatic agents do not alter the underlying neurodegenerative disease progression, and their efficacy reduces as the disease advances (Birks and Harvey 2006). On the other hand, while anti-Aβ antibodies regulate progression, their clinical benefit is modest rather than curative. Their broader utility is further repressed by the risk of ARIA, expensive, needed intravenous drug administration, and lower BBB penetration (van Dyck et al. 2023). Recent studies evaluated that AD pathogenesis contributes intertwined mechanisms including amyloid accumulation, tau hyperphosphorylation, neuroinflammation, synaptic decline, and oxidative stress single-target agents scarcely display comprehensive therapeutic control (Cummings et al.^ 2023^). There is therefore a strong rationale study for exploring alternative therapeutic strategies, specifically multi-target small molecules and drug delivery systems competent to addressing multiple central pathological molecular targets simultaneously. However, the magnitude of their clinical benefit remains limited, as they modestly slow cognitive and functional impairments rather than reversing or halting disease progression. AChEIs show symptomatic cognitive enhancement, improve cognitive function and activities of daily living (ADLs) (Fan et al. 2022). Traditionally, FDA-approved AD treatments—specifically AChEIs and memantine displayed strictly symptomatic treatment without changing the underlying of disease cascade (Birks and Harvey 2006). The therapeutic landscape has since evolved with the FDA approvals of anti-amyloid monoclonal antibodies, such as lecanemab and donanemab, which marked the arrival of disease-modifying therapeutic approach capable of clearing Aβ plaques from the AD patients (van Dyck et al. 2023; Sims et al. 2023). However, the clinical impact of these disease-modifying therapies remains a subject of active debate. While phase 3 trials evaluated statistically significant depletions in cognitive decline among early-stage AD patients, the absolute treatment effects are modest (van Dyck et al.^ 2023^; Sims et al. 2023). Additionally, their broader implementation is restricted by safety risks notably ARIA and expensive, requirement for daily basis MRI monitoring, and intravenous delivery demands (Knopman et al. 2021; van Dyck et al. 2023). Thus, while disease modification is now part of clinical practice, there demands immediate attention toward alternative small-molecule and multi-targeted therapeutic approach. The combination of memantine and donepezil provides the greatest benefit in case of moderate to severe AD patients (Singh et al. 2024). Anti-amyloid monoclonal antibodies may reduce amyloid plaques and prevent disease progression. The major limitation of current anti-Alzheimer’s drugs is that they primarily manage symptoms rather than halt or reverse disease progression (Ortiz-Islas et al. 2025). Therefore, alternative therapeutic strategies remain necessary because, despite decades of research, currently available treatments provide only modest clinical benefits. AChEIs enhance acetylcholine levels at the synapse and are referred to as cognitive enhancers. However, overdose may cause cardiovascular effects such as bradycardia and cardiac muscle abnormalities, as well as other adverse effects, including bronchorrhea, seizures, neuromuscular weakness, and paralysis (Thornton and Crane 2020). Memantine, which has low to moderate affinity for N-methyl-D-aspartate receptors (NMDARs), is sometimes considered less effective because it can inhibit physiological glutamate signalling necessary for normal synaptic function (Kaushik et al. 2026a; b). Its reported adverse effects include dizziness, confusion, dyspnea, and headache (Olivares et al. 2012). Aβ inhibitors may induce amyloid-related imaging abnormalities (ARIA), which include brain edema and microhaemorrhage, as well as other associated symptoms such as delirium, dizziness, confusion, and altered mental status (Ali et al. 2022). GV-971, an oligosaccharide-based drug extracted from marine brown algae, has been reported to improve cognitive performance in select clinical cohorts of patients with mild to moderate AD (Wang et al. 2019). AChEIs remain the first-line symptomatic treatment for mild to moderate Alzheimer’s disease. Clinical trials demonstrated modest but statistically significant therapeutic benefits, with mean ADAS-Cog enhancements of 2–4 points compared to placebo groups and cognitive improvements for 6–12 months. Recent studies have demonstrated slower cognitive deficits (0.2–1.4 points/year MMSE loss vs. 1.07–3.4 points/year untreated) and 27–42% reduction in mortality. These therapeutic drugs are recommended in major clinical guidelines and retain therapeutic relevance because of their identified efficacy, cheaper, and widespread availability. Common adverse effects such as nausea, diarrhea, and vomiting, that is typically dose-dependent and manageable through gradual dose reduction (Gao et al. 2024). Growing evidence reports that brain metabolic dyshomeostasis plays a significant role in AD progression. Alterations in cerebral glucose utilization and glucose uptake may precede cognitive decline by several decades, suggesting that brain hypometabolism plays an important role in Alzheimer’s disease pathogenesis. Furthermore, systemic metabolic disease, including obesity and type-2 diabetes (Malakar et al. 2026d), are well-established risk factors for AD. These several factors are linked with chronic neuroinflammation, mitochondrial impairment, and insulin resistance, all these parameters may heighten AD progression (Cen et al. 2025).

Comparative analysis indicates that PTP1B represents a more promising therapeutic target for AD than several other molecular targets currently under investigation. BACE1 inhibitors have largely failed in clinical trials due to psychiatric effects, severe cognitive decline, and hepatic toxicities (Egan et al. 2019; Hampel et al. 2021), GSK-3β inhibition carries a narrow therapeutic window and regulate Wnt molecular pathways (Lauretti et al. 2020), CDK5 shows paradoxical effects depending on disease stage (Liu et al. 2016), and both tau- and TREM2-directed approaches remain limited to targeting downstream consequences rather than the upstream drivers of pathology (Krasemann et al. 2017; Congdon et al. 2023), and demonstrate strong blood-brain barrier (BBB) penetration. PTP1B acts as negative regulator of IRS-1 and receptor tyrosine kinase receptor (Rahman et al. 2019). Preclinical evidence suggests that PTP1B inhibition offers a multi-target strategy capable of modulating central insulin signaling, reduces microglial Src/NF-κB-driven neuroinflammation, and indirectly normalizes overactive kinases such as GSK-3β, without the toxicity associated with directly knocking out GSK-3β catalytic activity (Combs et al. 2001). Taken together, this positions PTP1B inhibition as a more comprehensive, disease-modifying strategy than single-pathway approaches that have so far struggled to translate into clinical success.

PTP1B, also known as protein tyrosine phosphatase non-receptor type 1 (PTPN1), is a member of the protein tyrosine phosphatase (PTP) subfamily and plays a pivotal role in the negative regulation of insulin and leptin signalling pathways (Malakar et al. 2025b). It dephosphorylates insulin receptor substrates and downregulates the JAK2 signalling pathway (Zhao et al. 2022). PTP1B-null mice are resistant to obesity induced by either leptin gene deletion or a high-fat diet. The leptin signalling pathway has also been identified as a potential neuroprotective target in AD (Gomes et al. 2014; Carbone et al. 2024). PTP1B negatively regulates insulin receptor signalling and contributes to insulin resistance. Additionally, PTP1B directly regulates leptin receptors in hippocampal neurons (Cen et al. 2025). PTP1B deletion or genetic inhibition enhances insulin and leptin signalling and regulates glucose homeostasis in obesity and type-2 diabetes model (Malakar et al. 2026b). Moreover, these molecular pathways are associated with the modulation of cognitive impairment, dementia, and synaptic plasticity. PTP1B regulates tropomyosin receptor kinase B (TrkB) and nSOC pathways, both of which are linked to synaptic plasticity (Akhtar and Sah 2020). PTP1B also downregulates brain-derived neurotropic factor (BDNF) signalling by dephosphorylating TrkB (Ozek et al. 2014). In AD, PTP1B activity is further increased by proinflammatory mediators such as TNF-α and IL-1β present in cortical microglia (Pei et al. 1994) Fig. 1. Recent studies have revealed that PTP1B may act as a positive modulator of neuroinflammation (Song et al. 2016). PTP1B has emerged as an important positive regulator of neuroinflammation (Malakar et al. 2026d). In activated microglia, increased PTP1B expression stimulates Src kinase activation by dephosphorylation of its inhibitory tyrosine residue, resulting in potentiating NF-κB signalling and increased release of pro-inflammatory mediators including TNF-α, IL-1β, IL-6, iNOS, and COX-2 (Sharallah et al. 2026). Pharmacological inhibition of PTP1B diminished microglial stimulation and neuroinflammatory responses in both cell-based studies and animal models, demonstrating that PTP1B contributes to the progression of AD by inflammatory mechanisms (Song et al. 2016). However, several PTP1B inhibitors have been withdrawn from clinical trials due to limited efficacy, organ toxicities, and the large molecular weight of the compounds (Singh et al. 2022). Simufilam disrupts the pathological interaction between soluble Aβ42 and α7 nicotinic acetylcholine receptors (α7nAChR) by restoring the native conformation of filamin A. Filamin A abnormally connects to α7nAChR and several neuroinflammatory targets in AD, and Simufilam disrupts these pathological interactions (Wang et al. 2023a). Simufilam has also been shown to regulate insulin and leptin receptors signalling, reduce hyperactivation of the mTOR signaling pathway (Wang et al. 2023b). In AD preclinical models, Simufilam disrupts hyperphosphorylation of tau protein, Aβ aggregation, NFT lesions, production of neuroinflammatory cytokines, while restoring neuronal function and reducing synaptic degeneration (Wang et al. 2017). In a Phase I clinical trial, Simufilam was evaluated in healthy volunteers to assess its pharmacokinetic profile and safety. The study also demonstrated restoration of CSF and blood lymphocyte biomarkers (Wang et al. 2020). A succeeding 28-day placebo Phase-II clinical study result outcome in mild to moderate AD patients displayed improvement of CSF biomarker vs. placebo. The molecular design strategy aimed to retain the favorable CNS drug-like properties of Simufilam while introducing structural modifications capable of improving functional group interactions within the PTP1B non-competitive allosteric site, thereby providing a new avenue for the designing of potential disease-modifying Simufilam agents against AD. Additionally, the cell line studies reported that lupeol and betulinic acid reduce the PTP1B function enhanced by TNF-α activity in hippocampal neurons. Growing evidence reported that natural flavonoids isolated form the *Broussonetia papyrifera* plant demonstrated PTP1B inhibition (Chen et al. 2002). Although, quercetin-3-O-β-D-glucuronide and myricetin-3-O-β-D-glucuronide, extracted from *Cyclocarya paliurus* leaves displayed significant PTP1B inhibition with significant IC_50_ value 7.39 µg/mL and 9.47 µg/mL (Zhang et al. 2010).

**Fig. 1:**
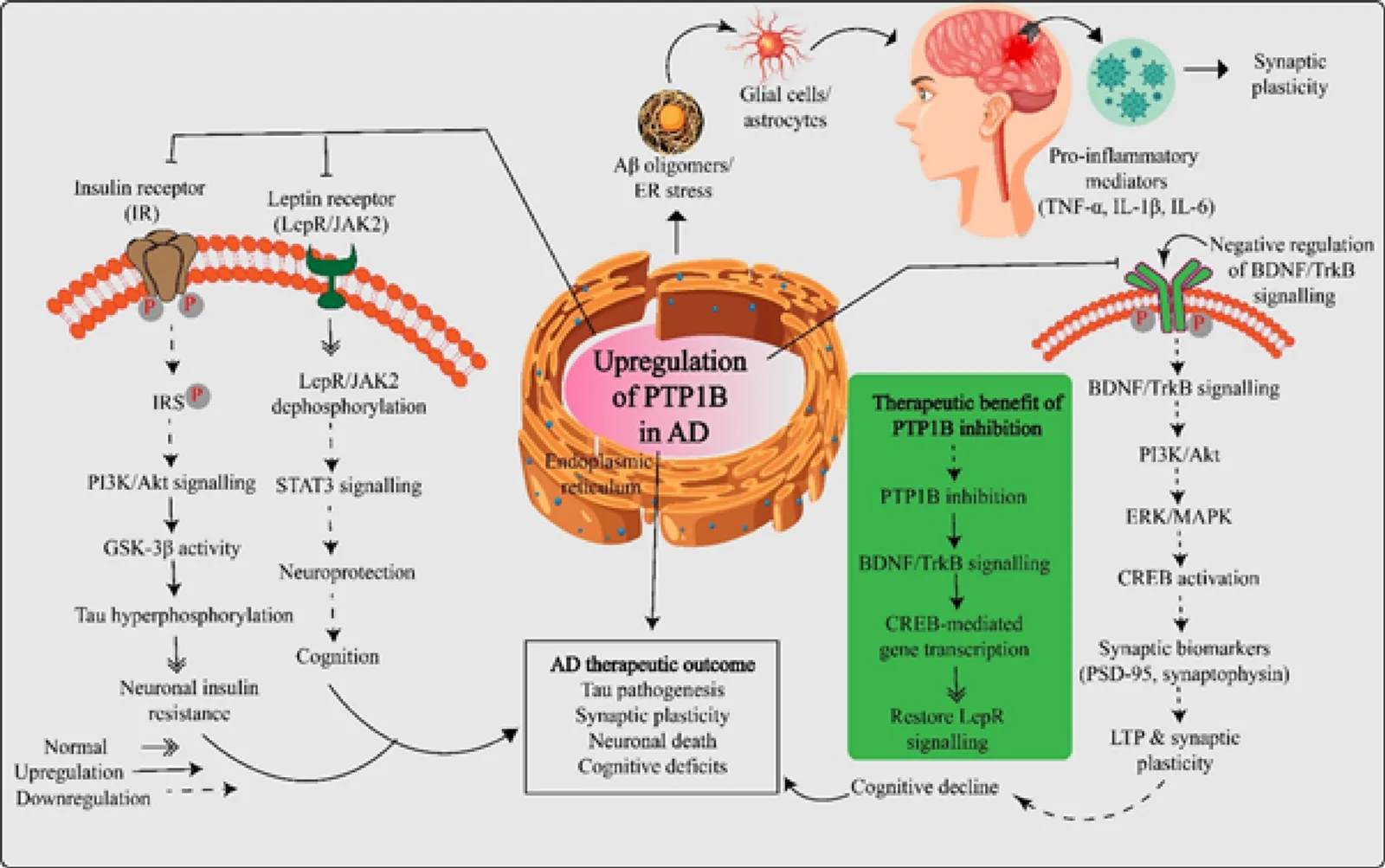
Schematic illustration of the role of PTP1B in Alzheimer’s disease. Aβ and ER stress-induced PTP1B stimulation disrupts insulin/leptin signalling pathways, potentiates neuronal degeneration, and inhibits BDNF/TrkB-mediated synaptic signalling, resulting in tau pathogenesis, synaptic plasticity, neuronal death, and cognitive deficits. PTP1B inhibition counteracts these pathological changes and shows neuroprotective effects

Computational studies may play a significant role in recognizing potent PTP1B inhibitors for diabetes, cancer, obesity, and AD therapy, hence further research is still needed to validate their therapeutic potential. These data highlight PTP1B as an important therapeutic molecular target in AD due to its contribution to synaptic dysfunction, neuronal loss, and metabolic overload (Patel et al. 2026; Jindal and Rai 2026; Abubakar et al. 2026). Simufilam, a drug developed by Cassava Sciences, was designed to target aberrant filamin A (FLNA) in AD and progressed to Phase III clinical evaluation (NCT05026177). Simufilam Phase III failure does not necessarily invalidate the underlying scaffold. The lack of clinical efficacy may be attributed to several factors including bioavailability limitations, off-target effects, and trial-related challenges rather than the scaffold itself (Srivastava et al. 2026). The trial was ultimately discontinued due to insufficient efficacy despite favourable safety and pharmacokinetic profiles. Based on the reported neuroprotective effects of Simufilam and the therapeutic relevance of PTP1B inhibition in AD, we hypothesized that rational structural modification of the Simufilam scaffold could yield novel allosteric PTP1B inhibitors with enhanced binding affinity, improved non-competitive allosteric-site selectivity, and favorable pharmacokinetic properties. We further anticipated that optimization of key pharmacophoric features would strengthen key molecular interactions with critical residues in the PTP1B non-competitive allosteric site, hypothesized to enhance predicted binding interactions and allosteric inhibition.

Given its compact, brain-penetrant spirocyclic scaffold, Simufilam represents a promising chemical starting point for the development of PTP1B inhibitors. Unlike previously reported PTP1B inhibitors, including the active-site inhibitors TCS401 and CPT157633 and the allosteric inhibitors Ertiprotafib and Trodusquemine (Kumar et al. 2020), Simufilam was originally developed for Alzheimer’s disease and possesses favorable CNS drug-like properties together with a chemically tractable scaffold suitable for structural optimization (Nand Rai 2026). Although active-site inhibitors often face challenges related to selectivity and pharmacokinetic properties, allosteric inhibition offers improved selectivity by targeting less conserved regulatory regions (Liu et al. 2022). These features support the rationale for selecting Simufilam as the parent scaffold for designing novel PTP1B-targeted therapeutics for AD.

In this study, 52 Simufilam derivatives were rationally designed through structural modifications at the R1 and R2 positions and evaluated as potential allosteric PTP1B inhibitors. Network pharmacology was employed to explore the therapeutic relevance of PTP1B, followed by molecular docking against the non-competitive allosteric site (PDB ID: 1T49) to evaluate binding affinity and key residue interactions. The lead compounds (A20, A26, and the co-crystal ligand) were further investigated using ADMET prediction, molecular dynamics simulations, PCA, MM/PBSA, DFT, and NBO analyses to assess their drug-likeness, binding stability, and electronic properties. This integrated computational workflow aimed to identify optimized Simufilam derivatives with improved binding characteristics and favorable pharmacokinetic properties as potential therapeutic candidates for AD.

## Materials and methods

### Rationale for structure-based drug design

Simufilam analogues designed with higher potency, efficacy, selectivity, and central nervous system (CNS) exposure, a systematic medicinal chemistry strategy was structured on the basic core 1,3,8-triazaspiro [4.5] decan-4-one scaffold (Wang et al. 2012). The central spiro core imposes a rigid 3D geometry that minimizes entropic penalty upon binding, while incorporating targeted steric bulk optimizes hydrophobic contacts within the active site (Carreira and Fessard 2014). The N-benzyl group at position 1 was maintained as an obligatory lipophilic anchor domain to keep baseline π-π hydrophobic amino acid interactions within the active binding pocket (Wang et al. 2012; Carreira and Fessard 2014). Central core optimization mainly focused on modifying the R_1_ and R_2_ positions: R_1_ Modification methods: The basic piperidine nitrogen functional group at R_1_ forms ionic bridge interact with conserved carboxylate amino acid residues in the active binding pocket (Wang et al. 2012). However, highly basic tertiary amines (pK_a_ > 9.0) often restrict passive BBB permeability and excessive hepatic clearance through cytochrome P450-mediated N-dealkylation (Pajouhesh and Lenz 2005; Wager et al. 2010). Substitutions at R_1_ moiety were structured to reduced amine basicity, tune lipophilicity (log D_7.4_ maintained between 2.0 and 3.5), and sterically designed the nitrogen functional group against metabolic degradation (Pajouhesh and Lenz 2005; Meanwell 2011). R_2_ changes Strategy: Derivatization at the imidazolidinone core R_2_ nitrogen was engineered to probe an adjacent, under-utilized binding pocket (Wang et al. 2012; Carreira and Fessard 2014). By assorting steric bulk and H-bonding capabilities at R_2_, we aimed to optimize complementary van der Waals residue contacts, increase molecular target affinity, and enhance selectivity over off-target receptors (Wager et al. 2010; Meanwell 2011).

### Screening of design compounds

**Fig. 2:**
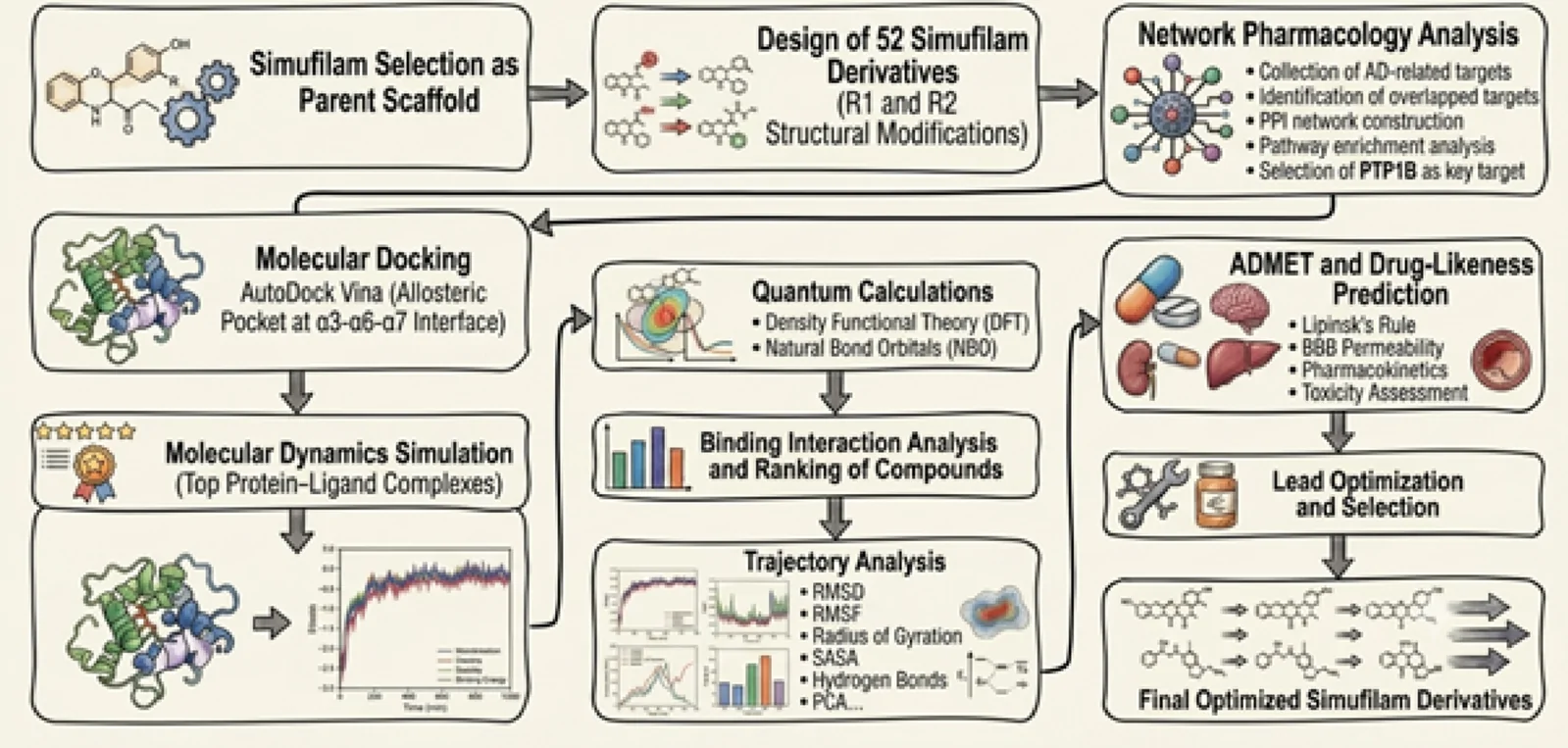
Illustrates schematic workflow of the computational study used in this research article

 In this work, a set of 52 Simufilam derivatives (SDs) (Table 1**)** were designed and evaluated for non-competitive allosteric site of the PTP1B enzyme (PDB ID: 1T49) enzyme. A library of 52 SDs was generated by introducing several substituents at the R₁ and R₂ positions while maintaining the parent compound. These positions were selected to enable structural diversification without altering the parent scaffold. Diverse substituents with varying steric, electronic, and hydrophobic properties, including alkyl, halo-substituted phenyl, methoxy-substituted phenyl, heteroaryl, and H-bond donor or acceptor functional groups, were incorporated to explore their impacts on predicted ligand–protein interactions. The molecular design was guided by computational evaluation by molecular docking and drug-likeness assessment. A rational, structure-based drug design strategy was adopted on target identification, as well as structure- and ligand-based strategies. These 52 derivatives were not arbitrarily selected, but were designed by a rational structure-based approach to explore key chemical variations around the parent compound while maintaining synthetic feasibility and enabling systematic comparison of predicted structure–activity relationship (SAR) trends. This designed library allowed us to balance novelty, chemical diversity, and synthetic feasibility, rather than generating an excessively large and less interpretable compound dataset. Figure 2. represents a schematic workflow summarizing the computational pipeline, including network pharmacology analysis, PTP1B target identification, Simufilam-based ligand design, molecular docking, molecular dynamics simulations, ADMET prediction, and lead optimization.

**Figure:**
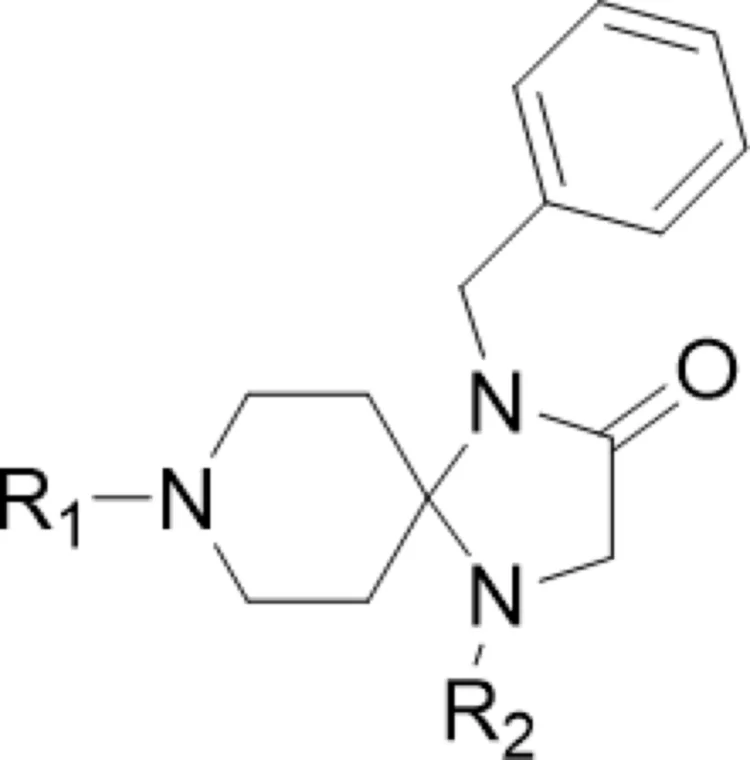
Basic Moieties of Simufilam

**Table 1:**
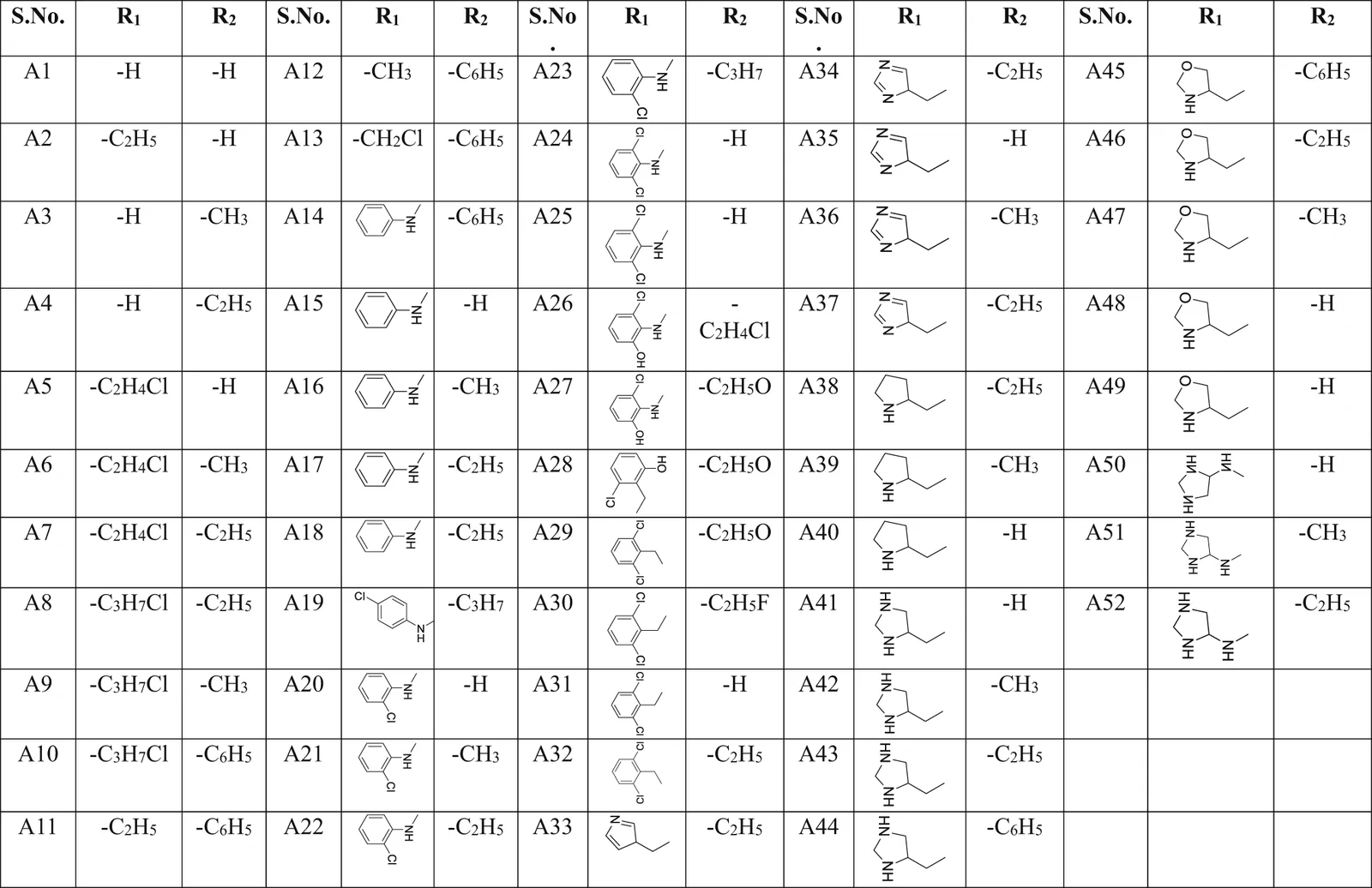
Simufilam drug design structures and their derivatives from A1 to A52

### Network pharmacology

Network pharmacology (NP) was conducted to analyze potential targets of designed compounds (A20, A26) and co-crystal with protein tyrosine phosphorylation-based targets. Targets with a prediction probability ≥ 0.1 were retained, duplicate entries were removed, and only human (*Homo sapiens*) proteins were included for further analysis. Protein-drug interactions of the 3 compounds correspond to 233 active targets associated with AD pathogenesis identified by the Swiss Target prediction (https://www.swisstargetprediction.ch/) online server, disease-related targets were downloaded from several open databases including DisGeNET (https://disgenet.com/), GeneCards (https://www.genecards.org/), and GeDiPNet (https://gedipnet.bicnirrh.res.in) (Grijaldo et al. 2023). The protein-protein interactions (PPI) were analysed by using the STRING database (https://string-db.org/) online server, target associated pathways were mapped out by Cytoscape 3.10.4 software. Overlapping targets between compound prediction sets and disease profiles were mapped using Venn diagrams. Top-ranked targets were screened out using node degree (total number of nodes bound with neighbours), betweenness (shortest path distance between node pairs), and closeness (shortest distance between node-node) (Malakar et al. 2025c).

### Selection of protein structure

Several researchers have focused on the development of potent drugs or pharmaceutical products for the treatment of AD and the mitigation of AD-related symptoms. Using X-ray crystallography and other tools such as cryo-electron microscopy (Cryo-EM), numerous co-crystal structures of AD-related genes have been identified and deposited in the protein data bank (PDB) (https://www.rcsb.org/) with specific PDB IDs (Ghahremanian et al. 2022). PDB ID 1T49 was selected for molecular docking studies because it consists PTP1B in complex with a known allosteric inhibitor, thereby providing a structurally validated non-competitive allosteric site. Compared with other known PTP1B crystal structures, 1T49 captures a ligand-induced conformation of the non-competitive allosteric site which is particularly suitable for the screening and optimization of allosteric inhibitors. The protein with PDB ID: 1T49 was downloaded and prepared using AutoDock software (http://autodock.scripps.edu/) software (Morris et al. 2009).

The preparation process involved the removal of unwanted chains, co-crystallized ligands, water molecules, and metal ions, followed by the addition of polar/non-polar hydrogens and Gasteiger charges. The prepared protein was then analysed with Discovery Studio Visualizer, which was used to identify the active catalytic binding pocket (Baroroh SSi, MBiotek, et al. 2023). For protein–ligand docking, the.pdbqt file was opened in the visualizer, and the ‘Define and Edit Binding Site’ tool was employed to determine the binding pocket. By using AutoDock Vina, molecular docking was performed using a grid box centered on the known non-competitive allosteric site of PTP1B located at the interface of helices α3, α6, and α7. This region, which encompasses the α7 helix regulatory site, was selected because allosteric inhibition of PTP1B has been shown to stabilize inactive conformations and provides improved selectivity compared with targeting the highly conserved catalytic site. The binding site 3-dimensional (3D) coordinates were defined as X = 45.90, Y = 17.90, Z = 15.17 to ensure site-specific docking coordinates. These coordinates corresponded to the active binding region for PTP1B (Malakar et al. 2025a).

### Molecular docking studies

Molecular docking analysis was performed using AutoDock software (Eberhardt et al. 2021). Docking was carried out on a library of 52 SDs. Initially, a compound library file was generated; throughout the configuration file containing the required docking variables for complex interactions was prepared (Ni et al. 2024). AutoDock Vina supports Perl scripting for automated docking, which facilitates protein and ligand preparation, molecular docking, dynamics simulation, and result analysis (Sarkar et al. 2024). The docking step was executed using a Vina Perl script, which integrates the configuration file via the command prompt (Aslam Mohd, Malakar, and Nand, 2025). Additionally, protein-ligand 2D interaction maps and docking validation, including the highest binding scores and RMSD values, were analysed using Discovery Studio (Valdés-Tresanco et al. 2020).

### Molecular dynamics (MD) simulation

MD simulations were performed using GROMACS 2020 (Makarewicz and Kaźmierkiewicz 2013; Abraham et al. 2015) to investigate the physical interactions of the ligand-protein complex in a biophysical environment. Topology files for the protein-ligand complex were generated using the CHARMM General Force Field (CGenFF), CHARM27, and Swiss Param (http://www.swissparam.ch/). All simulations were carried out in the Ubuntu Linux environment (Malakar et al. 2025c). The system was solvated using the TIP3P water model in a 1.0 nm triclinic box and neutralized with Na^+^ and Cl^−^ ions (Bjelkmar et al. 2010). Energy minimization was followed by equilibration under NVT and NPT ensembles at 300K and 1 bar, after which production runs were extended to 1000 ns. Simulation trajectories analysis was analysed using the GROMACS package, including root mean square deviation (RMSD) (Malakar et al. 2025a), root mean square fluctuation (RMSF), radius of gyration (Rg), hydrogen bond (H-bond) analysis, and ion pair interactions (Ishak et al. 2017). RMSD was calculated to assess equilibration and structural stability of the protein–ligand complex over 1000 ns (Law et al. 2005). RMSF was assessed to analyze the local complex fluctuations in atomic positions at the active residue level during the molecular simulation (Martínez 2015). Rg was analyzed to evaluate structural rigid compactness, where lower Rg values represent a higher compact complex, and higher values reflect denote increased flexibility (Lobanov et al. 2008). Solvent accessible surface area (SASA) and center of mass (CoM) were also evaluated at 1000 ns.

### Principal component analysis (PCA)

The MD simulation trajectory files were initially centered and transformed for PCA analysis using the GROMACS analysis tool. The analysis was conducted in two steps: construction of covariance matrix and diagonalisation of the resulting matrix (Malakar et al. 2025c). The “gmx covar” command was used to compute the covariance matrix and principal vectors, while the “gmx anaeig” command was applied to analyse the eigenvectors and calculate the PCA data. The orientation and dominant motions of the protein-ligand complex were described by the first two eigenvectors (EV1 and EV2), which represents the principal modes of motion (Roy and Anbarasu 2025). PCA was conducted on the Cα atoms of PTP1B using the MD trajectories. Before analysis, all frames were least-squares fitted to the reference structure to decrease overall translational and rotational native or complex orientations. The covariance matrix of Cα atomic positional fluctuations was evaluated to obtain the eigenvectors and eigenvalues demonstrating the collective motions of the native protein and complex. The conformational space during the simulation was evaluated by projecting the trajectories onto the first two principal components (PC1 and PC2) axis. 

### Molecular mechanics poisson-boltzmann surface area (MM-PBSA) analysis

The MM-PBSA method is a computational approach used to integrate MD simulations with the estimation of free binding energies of protein-ligand complexes. The results provide several energy components, including total binding energies (GTOTAL). The gmx_MMPBSA tool, implemented within the GROMACS package, was used to calculate the binding free energies. MM-PBSA calculations were performed after removing unwanted water molecules and applying periodic boundary conditions for PTP1B in complex with multiple SD-designed molecules. The molecular geometry of the complexes was further optimized through energy minimization using molecular mechanics (Roy and Anbarasu 2025). MM/PBSA calculations were conducted using the g_mmpbsa package. A total of 1000 snapshots extracted from the MD trajectory were used for free-energy calculation. The binding free energy was decomposed into van der Waals, electrostatic, polar solvation, and nonpolar solvation components. Polar solvation energies were computed using the APBS Poisson–Boltzmann solver, while nonpolar contributions were identified from solvent-accessible surface area (SASA). Entropic contributions were not included in the calculations, and the resulting MM-PBSA energies were used for relative comparison of complex binding affinities.

### Density functional theory (DFT) calculations

DFT calculations were performed using Gaussian 16 for ligands exhibiting the lowest binding energy. Geometry optimization and frequency calculations were carried out using the B3LYP method with the 6–311 + G basis set (Malakar et al. 2026c). Geometric symmetry was relaxed, and the quadratically convergent SCF method with default solvation parameters was applied. Additional DFT calculations were conducted at the B3LYP/6–31G* level using geometries optimized at the B3LYP/6–311G* with Gaussian 94 program modules (Medimagh et al. 2023). These calculations provided insights into intramolecular stabilization and molecular interactions that may contribute to the biological activity of the molecules (Shekarkhand et al. 2022).

## Results and discussions

### Network pharmacology

**Fig. 3:**
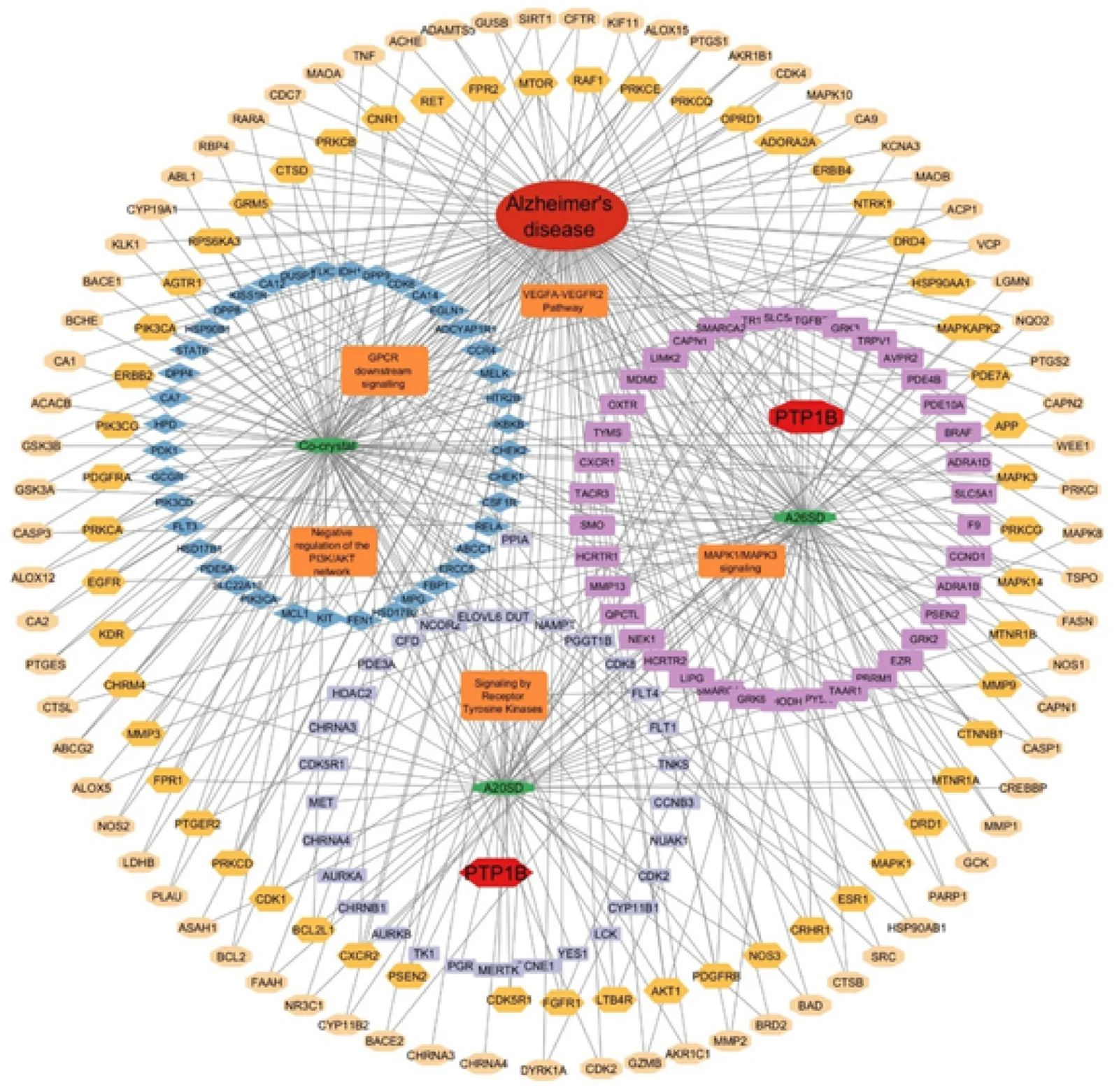
Schematic illustration of the molecular target of A20, A26, and the co-crystal. **A** Venn diagram of compound-disease-related common targets, **B** PPI of common targets, **C** common target of individual compound and single disease database, **D** compounds and multiple disease database analyze the tyrosine kinase signalling pathway (green color)

**Fig. 4:**
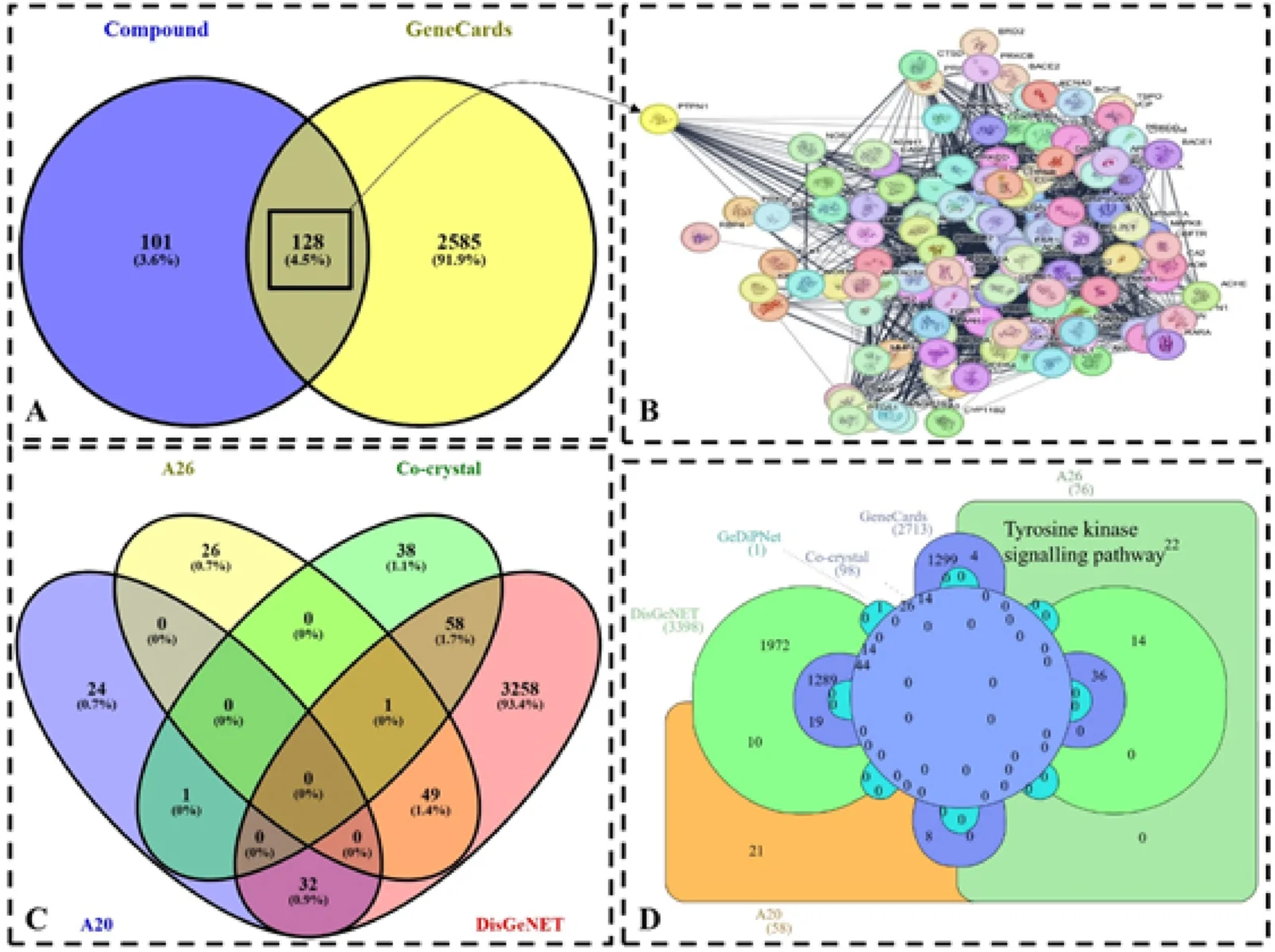
A complex network is designed between the compounds’ active network, 128 refined networks, target associate pathways network, a higher degree of nodes and edges-based actively involved targets with individual compounds

**Fig. 5:**
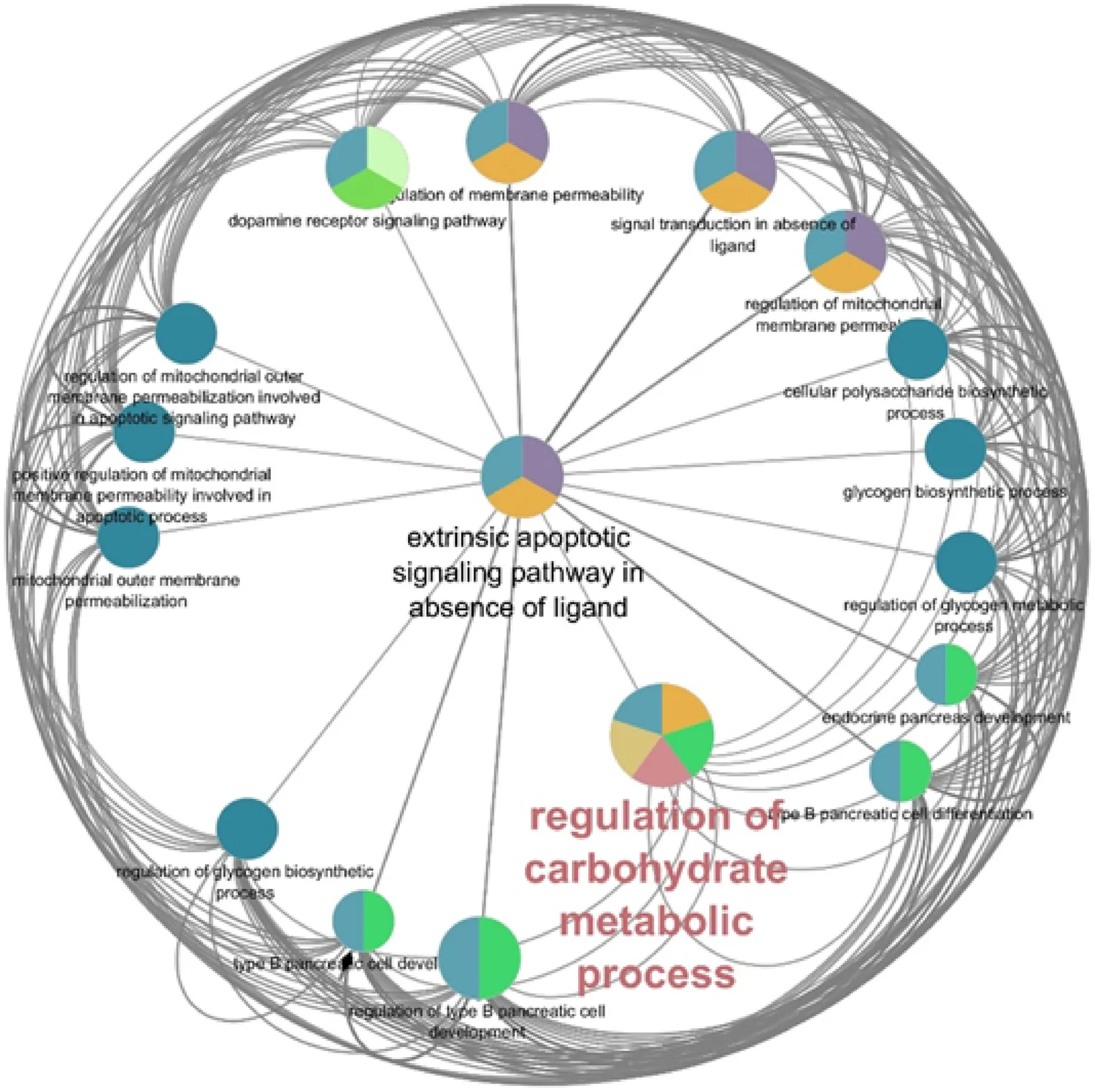
ClueGO was utilised to identify the target-associated distinct suitable biological pathways involve in the AD pathogenesis

**Fig. 6:**
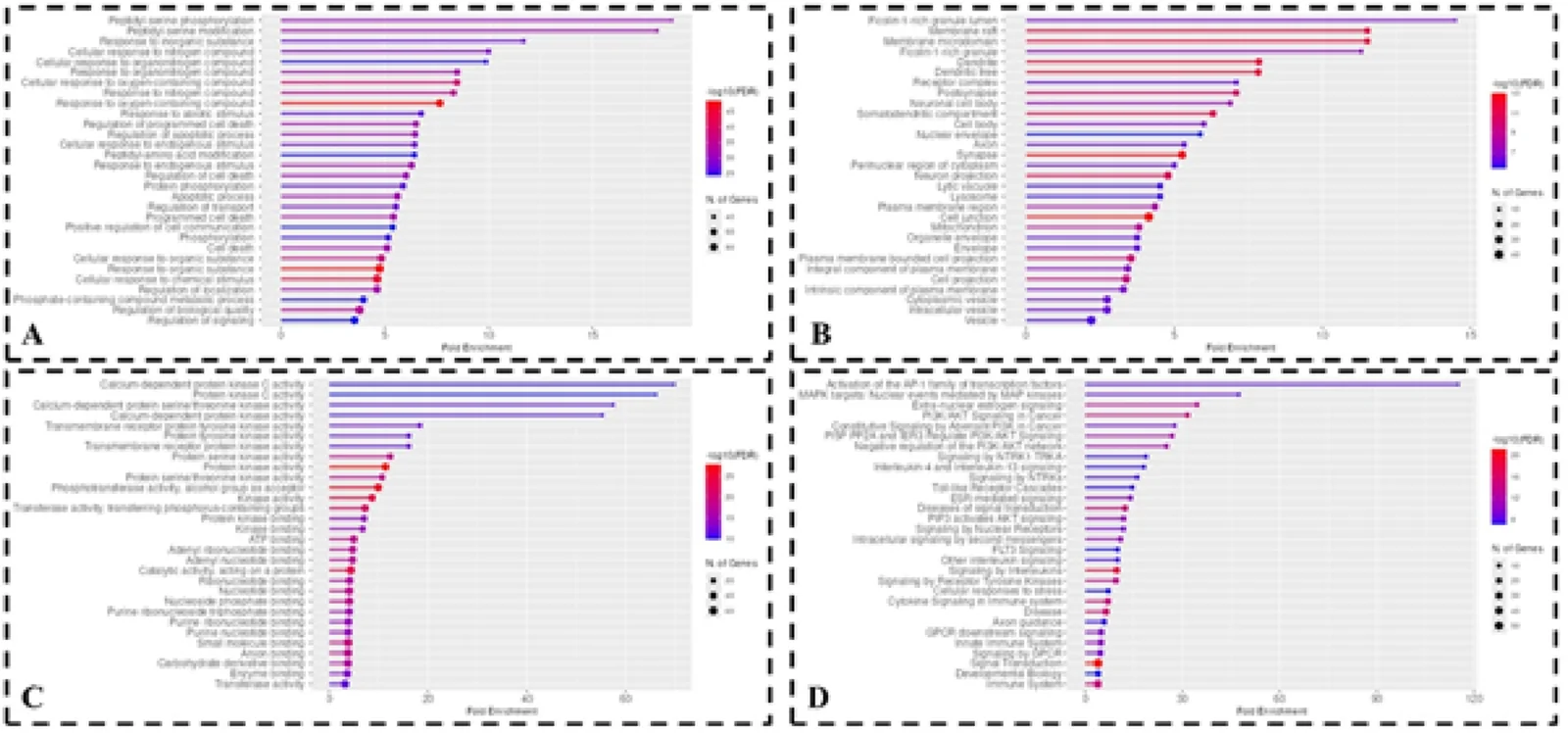
Lollipop graph plot of the top 10 BP, CC, MF, and Reactome pathways involved with compounds-disease-related active targets

A total of 2 SD-based designed compounds and a co-crystal were used for the design of NP due to these compound target PTP1B enzymes. The designed compounds A20, A26, and co-crystal were selected based on the molecular properties, including molecular weight, < 500 kDa, key targets, Lipinski rule of 5, number of H- bond donors and acceptors. The total number of potential targets of these compounds was 301; further duplicate targets were deleted and the refined targets were merged to examine 233 compound-based active targets. AD-related target retrieved from GeneCards (2713), DisGeNET (3398), and GeDiPNet (2919) Fig. 4C. By using a Venn diagram, SD-AD-related 128 common targets were examined and considered as potential therapeutic targets for AD treatment Fig. 4A. Furthermore, A20, A26, co-crystal, and different disease databases identified PTP1B potential targets among the designed SD compounds. The STRING database web server was utilised to identify the relationship between 128 active targets. Cytoscape 3.10.4 software was used for the analysis of the PPI of the molecular network. The PPI consists of 128 nodes and 1448 edges; the average node degree value was 22.6. Figure 4B, examined potent tyrosine kinase molecular signalling pathways for AD, Fig. 4D. The node denotes a single target, while the edge represents connections between more than two targets. Based on the degree, betweenness, and closeness, PTP1B, SRC, BCL2, AKT1, and CASP3 are considered as active key targets in the PPI molecular networks. A complex network consists of total compound targets, refined targets between AD and compound 128 actively associated targets, individual compounds associated refined targets based on higher nodes and edges value, and target associated top 5 molecular pathways including Signalling by receptor tyrosine kinase signalling pathways, negative regulation of the PI3K/Akt network, g-protein coupled receptor, vascular endothelial growth factor-A/vascular endothelial Growth Factor Receptor 2, mitogen-activated protein kinase-1/3 signalling Fig. 3. The ClueGO plugin was used for the identification of target-related carbohydrate metabolic molecular pathways that are involved in the pathogenesis of AD Fig. 5. To analyze the biological molecular mechanism of A20, A26, and co-crystal against AD, we conducted gene ontology (GO) enrichment analysis of 128 active therapeutic targets used in AD therapy. The GO analysis was divided into three groups including biological process (BP Fig. 6A), molecular function (MF Fig. 6B), cellular component (CC Fig. 6C), and Reactome pathways (Fig. 6D**)** plotted in lollipop format, false discovery rate (FDR) 0.05, red color represents highly active and blue color denote least active GO analysis pathway involve in the AD therapy. Among 128 active targets, PTP1B was selected based on the highest degree (92), betweenness (1969.41), and closeness (0.40) values. Moreover, we selected PTP1B as a potential therapeutic target for computational study.

### Molecular docking and its interacting residues

**Fig. 7:**
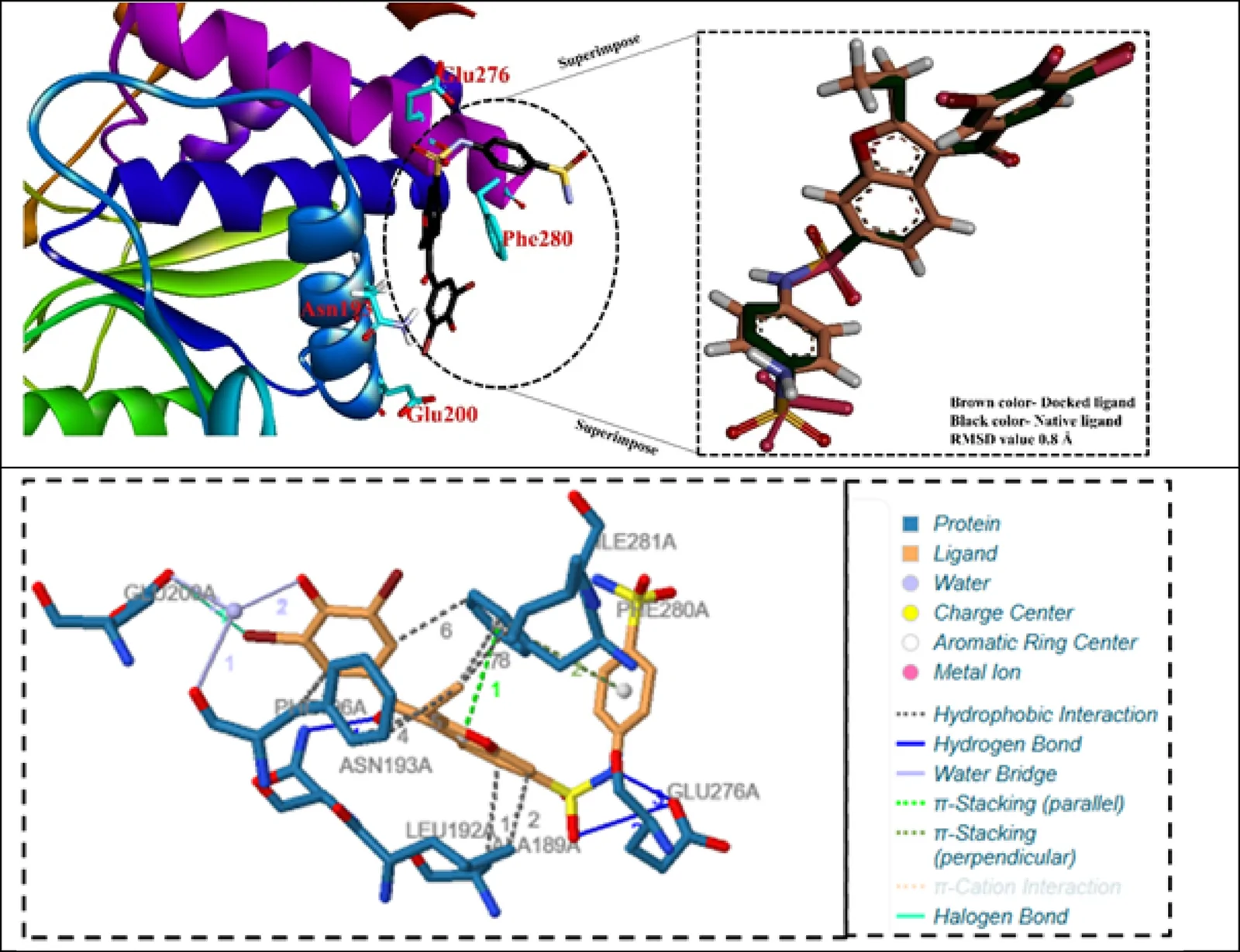
**A **Docking complex validation with an RMSD 0.8 and the active residues binding within binding pocket Asn193, Glu200, Glu276, and Phe280. **B **Detailed 3D interaction fingerprint mapped using the PLIP webserver.

 Molecular docking validation was performed using the AutoDock Vina tool Fig. 7. The co-crystallized ligand from PDB ID 1T49 was extracted and re-docked into the non-competitive allosteric site to validate the docking protocol. The superposition of the re-docked pose with the experimentally determined crystallographic pose yielded an RMSD of 0.8 Å, confirming the reliability of the docking methodology for reproducing the native binding orientation. Additional, non-covalent residues interaction patterns were cross-validated using the Protein-Ligand Interaction Profiler (PLIP) webserver. PLIP analysis demonstrated that the re-docked complex reproduced all key native non-covalent interactions, such as H-bonding, hydrophobic contacts, π-π stacking, halogen contacts, and water-bridge coordinates within the active binding pocket (Schake et al. 2025). To ensure the computational protocol reliably distinguished true active leads from non-specific binders, hit selection was governed by a multi-tiered consensus strategy. Designed Simufilam derivatives (SDs) (Table 2) were evaluated against the PTP1B non-competitive allosteric site, with calculated Gibbs free energy (ΔG, kcal/mol) reflecting thermodynamic complex stability. Ligand inhibition constants (Ki) were derived via ΔG = RTlnKi, where *R* = 1.987 cal/Kmol and T = 310.15 K (Jain and Bharti 2025). Among the 52 evaluated SDs, A20 and A26 emerged as the top-scoring leads, displaying favourable binding affinities of −10.8 kcal/mol and −9.5 kcal/mol, respectively (Table 2, Table 3). The significantly lower binding energy of A20 indicates a highly stable protein-ligand complex compared to A26. Both top candidates successfully engaged key residues across the catalytic and adjacent non-competitive allosteric sites, satisfying all consensus criteria and confirming their potential as selective inhibitors. Detailed 2D and 3D interaction diagrams for the co-crystal control (Figure S1A), A20 (Figure S1B), and A26 (Figure S1C) are provided in Figure S1. Given these challenges, recent research has shifted toward exploring both natural and synthetic scaffolds as sources of selective PTP1B inhibitors. Saeed et al. indicated that alkannin, a naturally occurring naphthoquinone compound derived from the Boraginaceae family, possesses PTP1B inhibitory activity with potential therapeutic relevance to diabetes. Computational analysis displayed favourable binding affinity for the complex of alkannin and PTP1B, with a binding energy of −8.1 kcal/mol. Key interacting residues included hydrogen bond contributors (Val297, Ala299, Leu300, as well as Ser302) and hydrophobic residues (Tyr20, Tyr48, Tyr79, Tyr111, Tyr219, Val47, Val297, Phe122, Cys298, Ala299, Leu300, and Leu301), which were identified in the protein-ligand complex. Biological evaluation result outcome showed that alkannin inhibits PTP1B with an IC_50_ value (19.47 µM). Enzyme kinetics revealed that alkannin exerts PTP1B and aldose reductase (AR) inhibition, denoting that it can bind not only with active residues but also with additional non-competitive allosteric sites of PTP1B (Saeed et al. 2024). Jin et al. identified targeting PTP1B with betulinic acid and lupeol, found that betulinic acid exhibited higher allosteric inhibition, with a binding affinity −12.2 kcal/mol and −10.6 kcal/mol for lupeol. The key interacting residues included hydrophobic amino acids (Ala189, Leu192, Phe196, Phe280, Trp291, and Leu294) and hydrogen bond-forming residues (Asn193, Glu276, and Gln288), which are located at the alpha seven (α7) helix. Biological result showed that lupeol and betulinic acid inhibited PTP1B with IC_50_ values 5.6 µM and 1.5 µM, respectively (Jin et al. 2016). These data are particularly important, as prior results have identified that the α7 helix as a regulatory non-competitive allosteric site of PTP1B (Choy et al. 2017a). Ligand binding at this region has been shown to regulate conformational alterations that may destabilize the WPD loop, hence suppress enzymatic function while provide higher selectivity compared to inhibitors of the highly conserved binding site. Although, previous reports have recognized the C-site, where compounds including difluoromethylphosphonic (DFMP) acid derivatives interact, as another different non-competitive allosteric site identified adjacent to the catalytic pocket that involves to selective enzymatic inhibition. Zhang et al., reported that DFMP acid derivatives, compounds 7 and 8, reduced PTP1B with binding affinities −11.74 and −9.06 kcal/mol, respectively. Key binding active residues, such as hydrophobic residues (Phe182, Ala217, and Ile219) and hydrogen bond residues (Lys36 and Arg47), identified at the C-site of PTP1B (PDB ID: 2QBP). Biological assay evaluated potent enzyme inhibition, with an IC_50_ value 7 nM for compound 7 and 90 nM for compound 8 (Zhang et al. 2017).

**Table 2:** Binding affinity of 52 SD derivatives and Co-crystal with PTP1B enzyme (PDB ID: 1T49)

CMPD	B.E. (kcal/mol)	CMPD	B.E. (kcal/mol)	CMPD	B.E. (kcal/mol)	CMPD	B.E. (kcal/mol)
1	−6.7	14	−8	27	−7.6	40	−7.5
2	−6.6	15	−7.9	28	−6.8	41	−7.5
3	−6.6	16	−7.4	29	−7.1	42	−7.1
4	−6.4	17	−7.2	30	−7	43	−6.7
5	−6.6	18	−7.2	31	−7.5	44	−7.5
6	−6.2	19	−7.7	32	−7.4	45	−7.8
7	−6.6	20	−10.8	33	−6.9	46	−6.9
8	−6.3	21	−7.2	34	−7.1	47	−6.8
9	−6.2	22	−7.1	35	−7.2	48	−7.7
10	−7.1	23	−7.4	36	−7.2	49	−7.2
11	−7.2	24	−7.6	37	−7	50	−7.6
12	−7.2	25	−8	38	−6.6	51	−7.2
13	−7.2	26	−9.5	39	−6.9	52	−7
53	Co-crystal		−6.0				

**Table 3:**
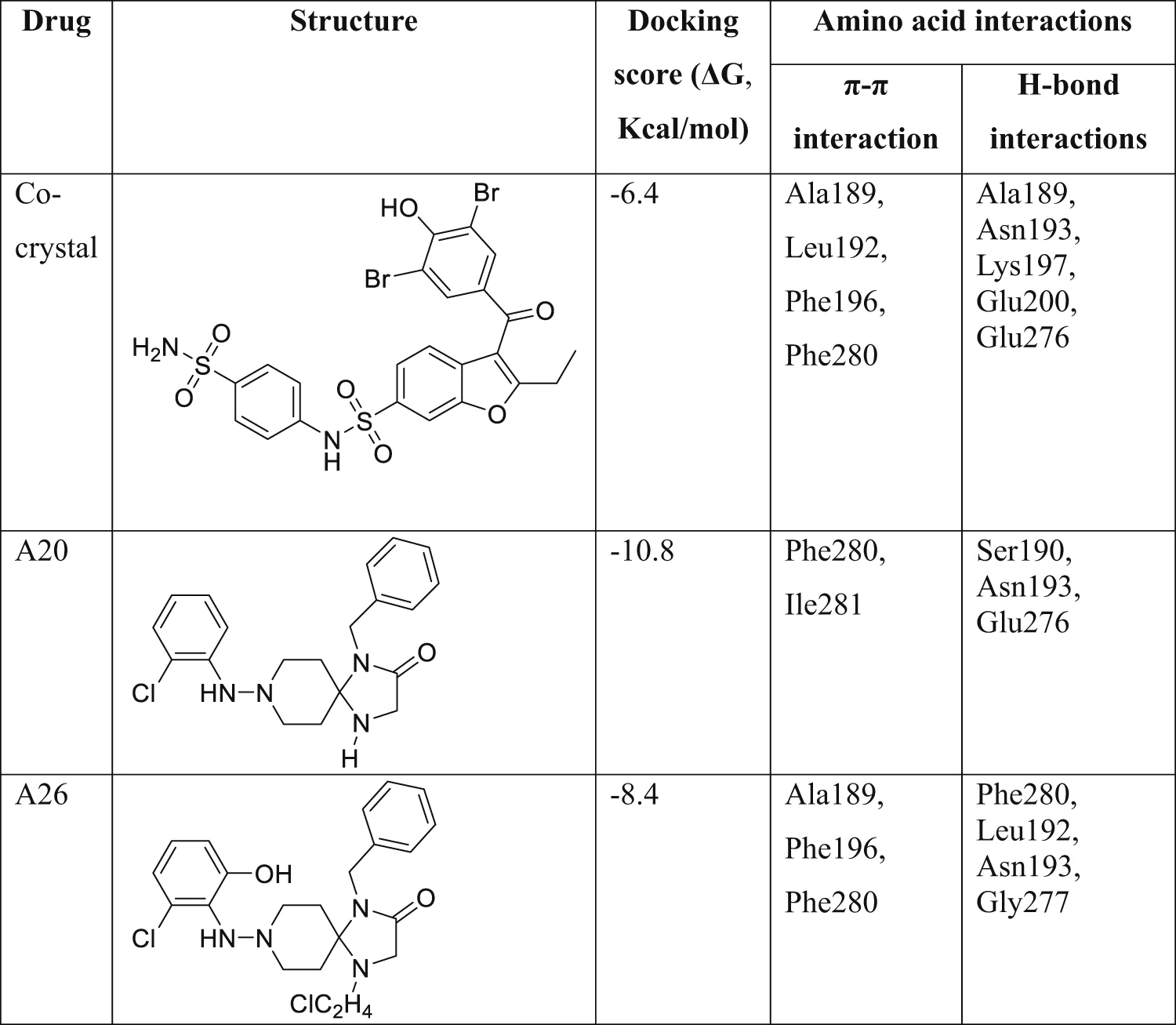
Binding affinity of the top 2 SDs derivatives and their amino acid interactions

### Mechanistic insights from protein-ligand interactions

Rationally designed SDs were molecular docked into the catalytic binding pocket of PTP1B (PDB ID: 1T49), revealing interactions that extend into non-competitive allosteric sites linked with the α3, α6, and α7 helices regions. These helices form part of the dynamic network which modulates the WPD loop regions, a structural element necessary for catalysis. The allosteric inhibitors examined in this study do not bind within the catalytic Tyr(P)-binding pocket. Instead, they bind to a separate non-competitive allosteric sites located mainly at the interface of helices α3, α6, and α7. Structural studies have displayed that binding at this catalytic site stabilizes the open, inactive conformation of PTP1B and attenuates the conformational changes required for WPD-loop closure, which is essential for catalytic activity. Thus, inhibition occurs indirectly through conformational regulation rather than through direct occupation of the catalytic site. Consistent with previous identifies by Choy et al. (Choy et al. 2017b), which displayed that conformational coupling within the α-helices and the WPD sites governs enzymatic function, the A20 compound was identified to regulate H-bond binding within this molecular network. Moreover, the H-bond binding of A20 with Asn193 and Glu276 are positioned in the structural network which couples α-helices to WPD sites mobility, rationalizing its identified ability to destabilize active loop closure (Table 3 and Figure S1B). Such changes are likely to differentiate the positioning of the catalytic residue Cys215 and destabilize WPD site closure, while impairing dephosphorylation. Additionally, inhibitor binding was found to restrict α3 helix rotation and alter the α7 helix, halt the formation of stabilizing H-bonds which normally favour a closed WPD loop conformation. In contrast, A26 found comparatively weaker interaction, but still occupied regions overlapping the catalytic and proximal non-competitive allosteric sites. A26 formed additional interactions with Leu192 and Gly277, active residues at the periphery catalytic binding pocket (Aslam Mohd, Malakar, and Nand, 2025), representing a weaker but broader engagement which may still perturb loop dynamics (Table 2 and Figure S1C). This dual involvement suggests that A26, while lesser potent, may retain inhibitory activity by partially perturbing WPD loop changes. Collectively, these outcomes suggest that A20 serves as a potent allosteric modulator by exploiting conformational cross-talk within the catalytic binding pocket and distal helical loop regions of PTP1B, while A26 acts as a supportive candidate with lower stability. These data and mechanistic deeper insights from molecular docking outcomes displayed the basis for carrying out further computational approach on A20 and A26, such as molecular simulations, PCA, MM/PBSA, and DFT analysis to strengthen and deepen the mechanistic insights of their inhibitory activity.

### Molecular dynamics simulations

MD is a real-time simulation technique commonly used to evaluate conformational alterations/changes, stability, and interactions within the protein-ligand complexes (Kharwar et al. 2025). Conformational stability up to 1000 ns allows atomic-level alterations in geometry and coordinates to be analysed (Esmaili and Shahlaei 2015). Simulations were carried out for 1T49-Co-crystal, A20 and A26 ligand complexes to assess comparative stability.

**Fig. 8:**
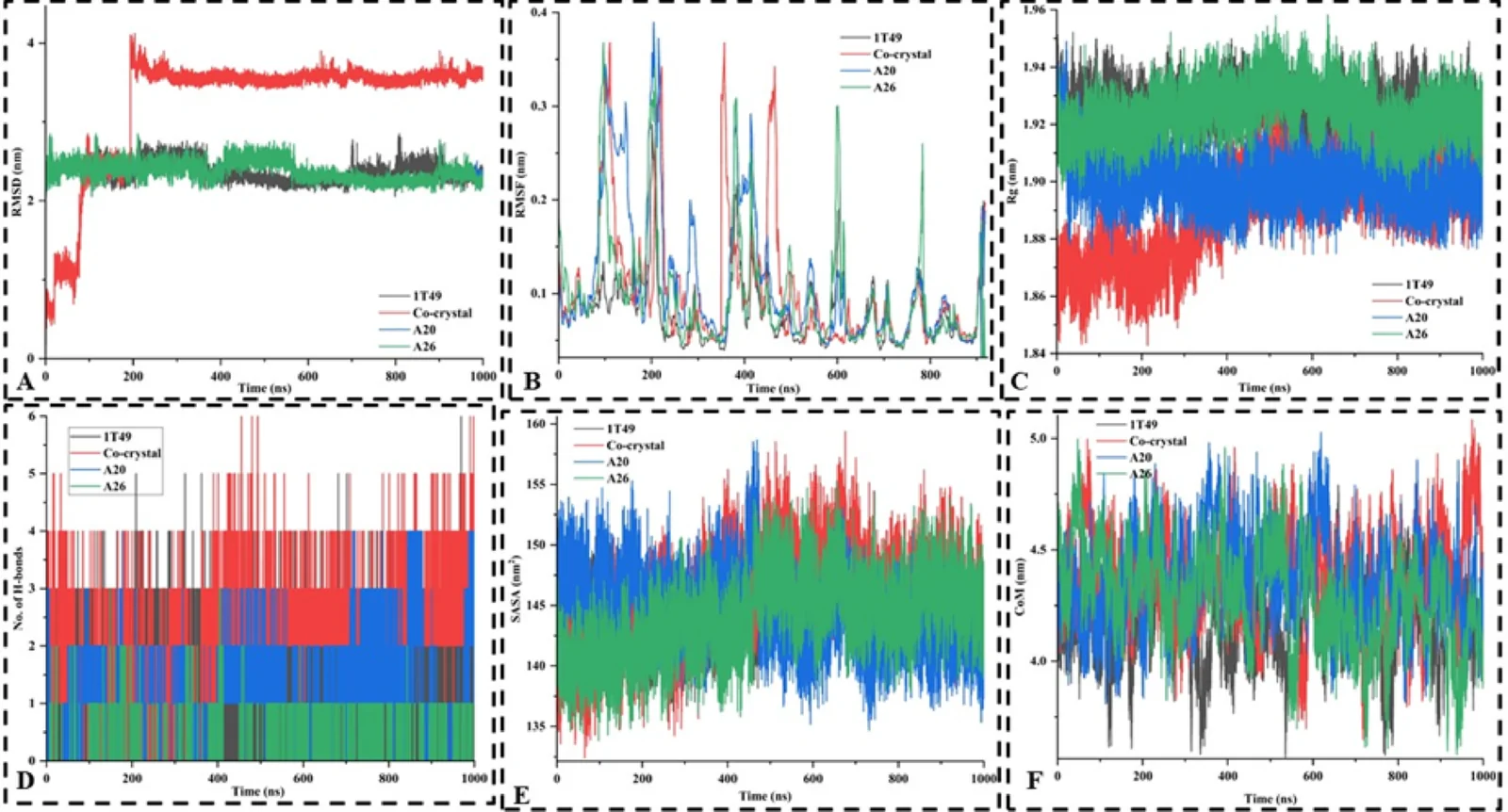
**A** RMSD, **B** RMSF, **C** Rg, **D** H-bond number, **E** SASA, and **F** CoM for Co-crystal-A20-A26 protein-ligand complex at 1000 ns

The RMSD graph of PTP1B bound with A20, showing favourable binding affinity, is illustrated in Fig. 8A. The RMSD profiles of the Co-crystal and A20 complexes demonstrated high stability, with fluctuations observed with values of 5.6 nm at 75 ns, 2.3 nm at 187 ns, 7.7 nm at 47 ns, and 2.2 nm at 217 ns. Further, the protein-A26 complex revealed higher deviations between 0 and 431 ns, with values of 8.6 nm at 89 ns and 0.4 nm at 431 ns, respectively; moreover, the complex is highly stable at 1000 ns time duration. The RMSF plot revealed the residue-level deviations points across the 1T49-Co-crystal, A20, and A26 complexes, which is illustrated in Fig. 8B. The higher fluctuations in the co-crystal complex identified 0.6378 nm (atom 4588) and 0.038 nm (atom 3709), while the A20 and A26 complexes remained comparatively lower fluctuations (Lemkul 2024). Rg was evaluated for all complexes (1T49-Co-crystal, A20, and A26), illustrated in Fig. 8C. The higher and lower Rg values (nm) were found as follows: 1.905 at 557 ns, 1.84 at 246 ns, 2.02 at 941 ns, 1.93 at 536 ns, 1.95 at 286 ns, and 1.88 at 764 ns. H-bond calculation revealed that a more than three H-bonds were stabled over the 1000 ns simulation (Malakar et al. 2026a), additional supporting the stability of all complexes (Fig. 8D). Further, SASA (Fig. 8E**)** and CoM (Fig. 8F**)** results were analyzed to evaluate binding stability and examined the surface area of the complexes. Both SASA and CoM values remained stable over the 1000 ns simulation for the 1T49-Co-crystal, A20, and A26 complexes, revealing consistency with the native protein (1T49).

### Principal component analysis (PCA)

PCA analysis was performed to examine the protein orientations influenced in the presence of ligand, which helps to interpret the confirmation from the dynamic trajectories. This analysis displays in-depth mechanistic insights in case of complexes and how they evolve over time duration and evaluates the most significant orientation patterns. Each point on the PCA graph denotes a snapshot from each simulation trajectories, projected on the first two principal components (eigenvectors) axis, which reveals the most stable orientations.

**Fig. 9:**
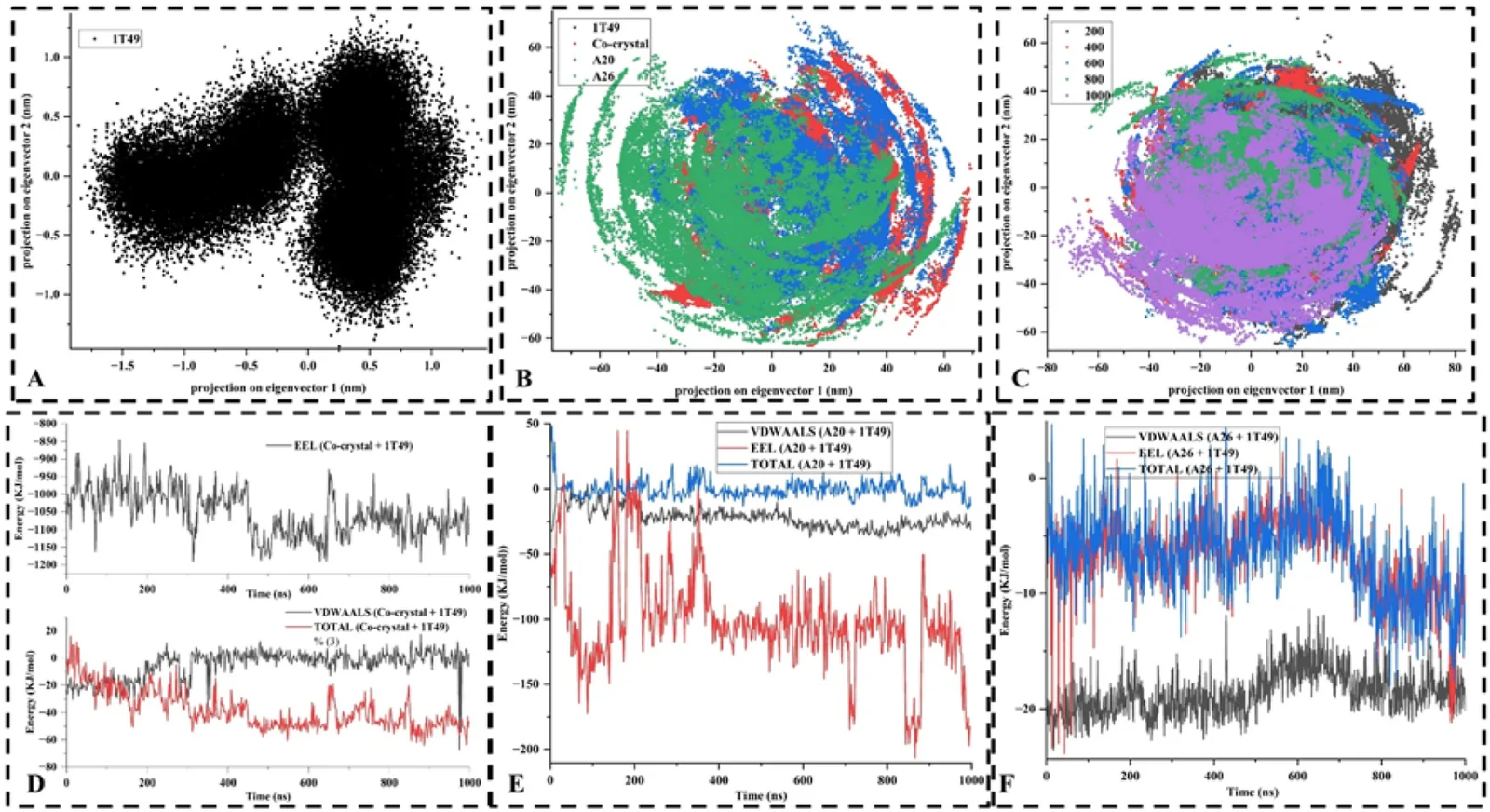
PCA analysis of **A** native protein (1T49), **B** Co-crystal-A20-A26 protein-ligand complex, **C A20** at different time interval like 0, 200 ns, 400, 600, 800, and 1000 ns, and, MM/PBSA calculation of **D** Co-crystal, **E** A20, and **F** A26 MM/PBSA of Co-crystal-1T49 complex

As displayed in Fig. 9A, the 1T49 occupies a minimal phase space within a molecule cluster, representing limited conformational alterations and higher complex stability without ligand. PCA results outcome further displayed that ligand binding poses enhances the phase space, making the 1T49 more flexible. Despite this increased flexibility, some variations in phase space were still observed across the complexes. For the 1T49-Co-crystal, A20, and A26 complexes, ligand binding significantly expanded the conformational space explored by the protein, as reflected in the broader distribution along the principal components, as shown in Fig. 9B. The A26 complex primarily moved along EV2, reaching a maximum value of 53 nm, whereas A20 complex was oriented along EV1, with a maximum value of 72 nm. The 1T49-A20 complex displayed intermediate behaviour in terms of phase space, structural changes, and stability (Kumari et al. 2021). PCA values calculated over 200 ns intervals for the 1T49-Co-crystal, A20, and A26 complexes, covering the entire 0–1000 ns simulation, are illustrated in Fig. 9C, Figure S2D, and S2E. These analyses represent the orientation of each complex over 200 ns and the movement along EV1 and EV2.

### Molecular mechanics poisson-boltzmann surface area (MM-PBSA) calculation

MM-PBSA calculations were performed to evaluate the binding energetics of protein-ligand complexes 1T49-Co-crystal, A20, and A26. The binding free energy was computed over a 1000 ns MD simulation. The calculations included contributions from VDWAALS, EEL, and the GTOTAL. The binding free energy results for the 1T49-Co-crystal, A20, and A26 complexes (Figs. 3E and 9D, and 3F) are as follows: for 1T49-Co-crystal, VDWAALS = −80 kJ/mol, EEL = −1150 kJ/mol, GTOTAL = −80 kJ/mol; for A20, VDWAALS = −28 kJ/mol, EEL = −200 kJ/mol, GTOTAL = −21 kJ/mol; and for A26, VDWAALS = −23 kJ/mol, EEL = −20 kJ/mol, GTOTAL = −25 kJ/mol.

### Analysis of pharmacokinetic parameters

Pharmacokinetic parameters are a fundamental aspect of drug discovery, as they help analyse the biological activity and behaviour of compounds. Lipinski’s Rule of Five was applied to identify the drug-likeness of the compounds (Vijayakumar et al. 2020). The physicochemical properties of Co-crystal, A20, and A26 compounds among the SDs were analysed by using Schrodinger suite (Qikprop module) 2023-25. The ADMET properties of the A20 and A26 compounds were also evaluated for a better understanding of their pharmacokinetic profiles, with results given in Table 4. A20 does not violate the molecular weight criterion (MW = 370 Da < 500 Da), H-bond donor criterion (4 donors < 5), or lipophilicity criterion (QPlogPo/w = 1.74 < 5). The observed RO5 violation arises from the H-bond acceptor count, which was identified as 12.2, more than the recommended threshold of 10 acceptors. Consequently, A20 exhibits a single Lipinski violation (RO5 = 1). Simufilam is an orally active drug candidate, brain-penetrant small molecule mainly developed for AD therapy. The compound shows favorable physicochemical properties for CNS exposure and has been revealed to restore the native molecular conformation of altered filamin A protein, thereby normalizing several molecular signalling pathways linked with synaptic plasticity, neuronal death, and Aβ toxicity. Its demonstrated ability to penetrate the BBB, together with its clinical advancement and neuroprotective activity, makes Simufilam an attractive lead basic scaffold for the design of potent derivatives. Accordingly, Simufilam was chosen as the parent scaffold for the designing of allosteric PTP1B inhibitors aimed at potentiating therapeutic efficacy against AD (Srivastava et al. 2026).

**Table 4:** Physicochemical properties of co-crystal, A20, and A26 SDs derivatives

Compound	MW (g/mol)	CNS	donorHB	accptHB	QPlogPo/w	QPlogBB	RO5
Co-crystal	658	2	2	6.5	2.28	0.65	0
A20	370	−2	4	12.2	1.74	−3.00	1
A26	449	1	2	7.2	2.8	0.4	0

### Density functional theory

**Fig. 10:**
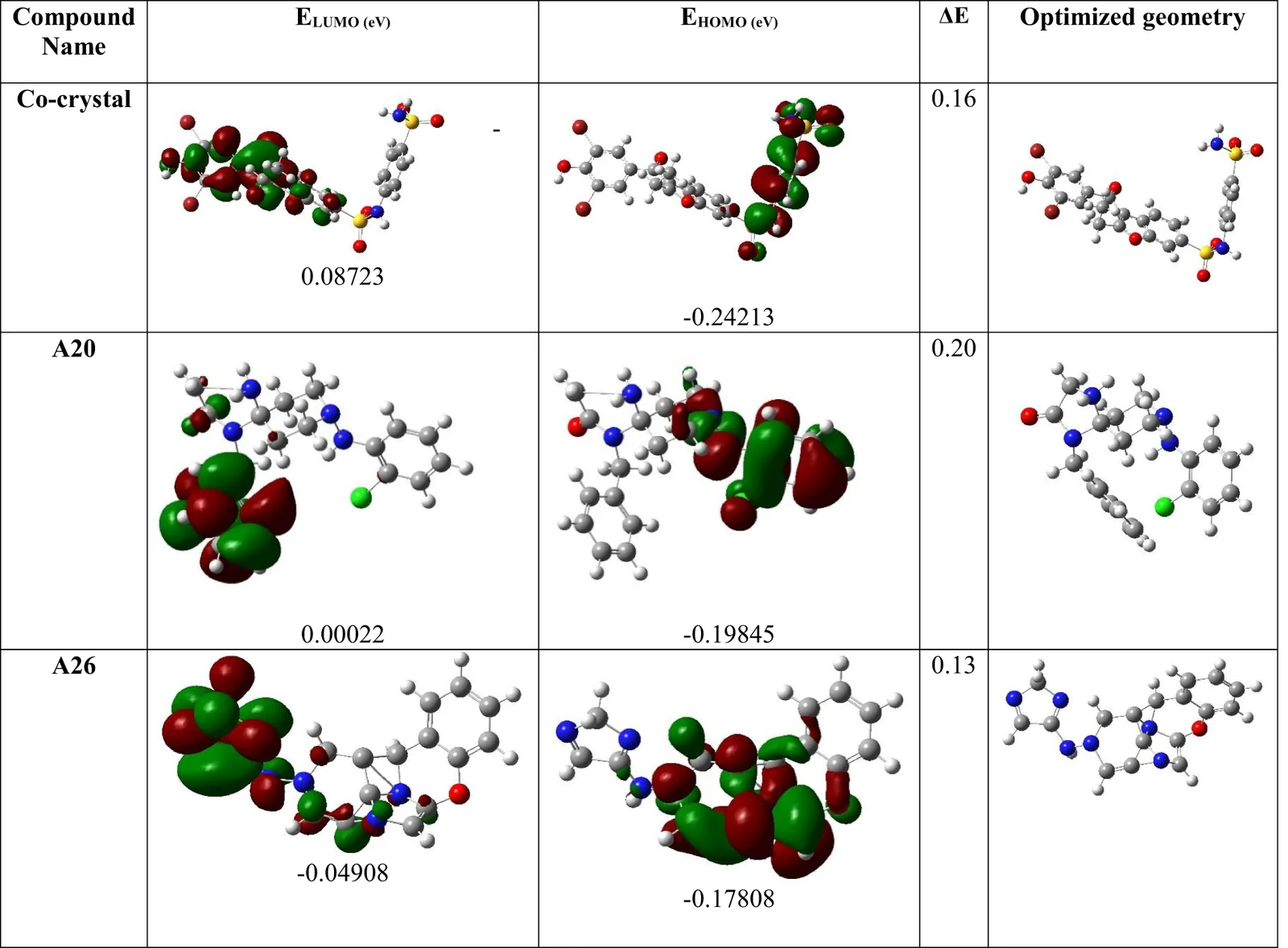
Diagrammatic illustration of HOMO, LUMO energies, and optimized geometry of 1T49-Co-crystal, A20, and A26

The DFT calculations were carried out to complement docking and molecular dynamics analyses by providing electronic structure and thermodynamic insights into the ligands and their interactions. Initially, three systems, the co-crystal, A20, and A26, were evaluated to determine their stability, polarity, and optimized geometries. The molecular orbitals and energy profiles are mentioned in Fig. 10. Thermodynamic energies, such as zero-point, thermal, enthalpies, free energies, optimization energies, and dipole moments, are mentioned in Table 5. The dipole moment results denoted that A20 (4.39 D) is highly polar than A26 (3.74 D) but lower polar than the co-crystal as a reference compound (11.54 D). This polarity values may.

 change solvation nature and binding confirmation. Such molecular descriptors, with the orbital energies, assist in assessing compound thermal stability and polarity in optimized geometries (Al-Adilee et al. 2024). Due to their highly precision and reliability, DFT-related quantum analysis remains a widely used computational method for the calculation of electronic energies and molecular structures (Abu-Izneid et al. 2024). Among the ligands, A20 was prioritized for deeper residue energy calculation because, in addition to exhibiting favourable electronic polarity, its molecular docking outcome revealed consistent and strong molecular interactions with the active binding site residues (Tyr46, Ser216, Asp193, and Glu276), making it the most promising therapeutic candidate for quantum calculation. Frontier molecular orbitals, including the HOMO (highest occupied molecular orbital) and LUMO (lowest unoccupied molecular orbital), were displayed to provide mechanistic insights into reactivity and electronic configurations (Huq et al. 2024). The HOMO denotes the highest-energy orbital consisting electrons and serves as an electron donor, while the LUMO denotes the lowest-energy orbital and serves as an electron acceptor (Raksha et al. 2025). The calculated HOMO, LUMO, and optimized geometry of A20 are mentioned in Table S3. The HOMO–LUMO electronic gaps are on the order of ~0.19–0.21 Hartree (mentioned as −0.19 to −0.21 Hartree in the table because E_HOMO_ < E_LUMO_), corresponding to ~5.4–5.9 eV. Among these, Tyr46 displays the smallest electronic gap (~5.43 eV), representing slightly more electronic softness/reactivity relative to the others, however Asp193 and Glu276 have slightly more gaps (~5.8–5.9 eV). These electronic properties are consistent with the active residues reported in ligand recognition in our docking result analysis. Finally, the dipole moment (µ) of A20 was also implicated for evaluating compounds polarity and the distribution of total charge among the compound and its interacting active binding pocket residues. Polarity may also alter solubility in the present of different solvent (Quayum et al. 2024).

**Table 5:** Quantum energy calculation and dipole moment energy of A20, A26, and Co-crystal

Compound	Zero-point energies	Thermal energies	Thermal enthalpies	Thermal free energy	Optimization energy	Dipole moment (Debye)
	(Hartree/Particle)					
Co-crystal	−7430.29	−7430.26	−7430.26	−7430.37	−7430.68	11.536484
A20	−1522.43	−1522.41	−1522.41	−1522.49	−1522.85	4.392040
A26	−1052.88	−1052.86	−1052.86	−1052.93	−1053.16	3.738659

### Natural bond orbital (NBO) analysis

The NBO calculation was conducted to gain mechanistic insights into the electronic configuration and bonding nature of the compounds. This calculation requires for studying symmetric and asymmetric molecules nature, as it displays accurate molecular insights on aromatic systems, specifically those consisting heterocyclic ring moieties (Shekarkhand et al. 2022). For the proposed A20 compound, NBO analysis was used to examine the molecular energy gap among the HOMO and LUMO, and to evaluate its residues interactions within the binding pocket, such as Tyr46, Ser216, Ala217, and Thr263. The data of this calculation are depicted in Figure S3.

NBO calculation of A20 displays a molecular network of intramolecular H-bonds (O/N···H distances 1.732–1.772 Å, with a lower contact at 2.107 Å) that pre-organizes the molecule into a compact, H-bond-stabilized orientation; a standard C–Cl bond length (1.719 Å) is also identified. The dominant donor–acceptor residues interactions are n(O/N) σ(N–H/C–H)* hyperconjugation and π-π* delocalization across carbonyl functional groups and aromatic ring fragments, which collectively stabilize the optimized compound geometry and increase electron delocalization. These properties are consistent with the frontier molecular orbital gap depicted for A20 (~5.1–5.2 eV) and its moderate dipole energy. In summary, NBO calculations indicate that intramolecular hydrogen bonding and donor–acceptor delocalization play a significant role in stabilizing A20’s molecular geometry and enhancing electronic delocalization, energetically favouring a pre-organized conformation for site binding. To contextualize the medicinal chemistry value of compound A20, its structural and predicted pharmacodynamic properties were compared against established PTP1B allosteric inhibitors reported in the Table 6.

**Table 6:** Comparative analysis of A20 vs. benchmark PTP1B allosteric inhibitors

Basic moiety	Compound	Active binding pocket	MW (g/mol)	Predicted or examined BBB permeability	Result outcomes	Limitation	References
Benzofuran or indole derivatives	Compound 2	α3/α6/α7 Allosteric sites	480–550	Low to moderate	IC50 ~ 350 nM	TPSA > 110 Å limited CNS penetration	(Wiesmann et al. 2004; Friedman et al. 2022)
Aminosterols	Trodusquemine (MSI-1436)	C-terminal non-catalytic site	628.9	Very Low	Examined in vivo metabolic & neuroprotective effects	High MW (> 600 Da) & charged polyamine tail prevent direct BBB crossing	(Krishnan et al. 2014)
Polyamine Alkaloids	Claramine	C-terminal Allosteric Pocket	425.7	Moderate	Restores central insulin signaling without targeting active site	Potential off-target ionic toxicity; high basicity (pKa > 10)	(Qin et al. 2015)
Spiro-Hydantoin Derivative	A20	α3/α6/α7 Allosteric sites	355.9	High (CNS MPO > 4.5, logD_7.4_ = 2.48	Rigid 1,3,8-triazaspiro core; balanced lipophilicity; uncharged basic center optimized for BBB penetration	Requires further in vitro enzymatic validation	

## Conclusion

This in silico study revealed that the structural dynamics and binding affinity of designed compounds of Simufilam derivatives (A1–A52) targeting PTP1B as potential computational leads for AD therapy. The PTP1B molecular target analysed using network pharmacology to identify molecular interactions with active biological therapeutic targets. To explore feasible therapeutic benefits in preventing Alzheimer’s disease (AD) symptoms, with a series of Simufilam drug derivatives (A1–A52) was designed. Among these derivatives, A20 revealed the stronger molecular binding affinity (−10.8 kcal/mol) toward the PTP1B enzyme, highlighting its favorable computational ranking relative to other derivatives in the compounds. Molecular docking and simulations further examined that the PTP1B-A20 complex exhibited the highest binding stability at the non-competitive allosteric sites of PTP1B, while most of the other SDs derivatives induced conformational changes within the native protein (1T49). Remarkably, A20 maintained a maximally exposed conformation changes that reinforced its stable binding drug profile. PCA calculation displayed that PTP1B significant conformational flexibility in the absence of A20, enabling traversal of a broad conformational landscape. In contrast, simulated interactions with A20 were associated with reduced conformational flexibility, yielding a more compact calculated ensemble. Importantly, A20 residue interactions were identified to initiate higher conformational re-organization in the protein sequence, representing a profound stabilizing changes at the binding molecular interface. To elucidate the non-competitive inhibition mechanism of compound A20, its binding interactions were contextualized within the established allosteric regulatory framework of PTP1B (Wiesmann et al. 2004; Friedman et al. 2022). Catalytic activity in PTP1B requires dynamic motion of the flexible WPD loop (residues 177–185), which must transition from an “open” state to a “closed” conformation to correctly position catalytic Asp181 relative to active-site P-loop (Cys215–Arg221) for substrate cleavage (Brandão et al. 2012; Friedman et al. 2022). Computational docking demonstrates that compound A20 occupies a hydrophobic C-terminal non-competitive allosteric site situated approximately 20 Å from the active site, delineated by helices α3, α6, and α7 (Wiesmann et al. 2004). The o-chlorophenyl hydrazine tail at R_1_ position establishes hydrophobic contacts and halogen interactions with Leu192, Ala189, Phe280, and Leu281, while the spiro-hydantoin core anchors the compound within the surrounding helical framework. Occupancy of this site by A20 prevents the C-terminal α7 helix from adopting the packed, ordered conformation required to facilitate closure of the flexible WPD loop (Wiesmann et al. 2004; Krishnan et al. 2014). By locking the WPD loop in its open, inactive state, A20 decouples substrate binding from enzymatic cleavage, leading to a reduction in catalytic rate turnover (K_cat_ reduction). Because this non-competitive allosteric site is distinct from the highly conserved, positively charged active site, targeting this region provides a structural basis for high isoform selectivity and improved cellular permeability (Tonks 2006; Krishnan et al. 2014). Complementary DFT calculation further examined the electronic configuration of A20, reveals its higher electronic reactivity, stability, and polarity in association with active residues such as Tyr47, Ser216, Asp193, and Glu276. NBO calculation additionally revealed that intramolecular H-bonding and donor–acceptor delocalization involves to A20’s pre-organized and energetically suitable binding geometry. In silico ADMET drug profiling examined that A20 exhibits favorable blood-brain barrier permeability and solubility, it also exhibited certain molecular violations of Lipinski’s rule parameters, warranting further geometry optimization. Although the present study employed an integrated computational method contributing network pharmacology, molecular docking, molecular simulations, principal component analysis, MM/PBSA, density functional theory, natural bond orbital, and ADMET prediction, several limitations have been acknowledged. The predicted binding affinities, interaction patterns, and pharmacokinetic properties are based on theoretical models and may not fully reflect biological behavior under physiological conditions. Furthermore, molecular docking and molecular simulations provide insights into protein–ligand interactions but cannot directly establish biological function, target selectivity, or therapeutic efficacy. Future development of A20 relies on systematic lead optimization. Synthetically accessible through a simple and concise two-step synthesis process, A20 targets the non-conserved α3/α6/α7 allosteric sites, providing a scaffold to guide target selectivity over homologous TCPTP in future experimental studies to prevent off-target effects. To improve metabolic half-life and reduce P450-mediated oxidation of the hydrazine cross-linker, bio-isosteric modifications (-CH_2_NH-, triazoles) will be examined. Additionally, formulating A20 into PLGA nanoparticles will enhance aqueous solubility and facilitate CNS drug delivery for upcoming in vitro and in vivo studies. However, the identified Simufilam derivatives should be considered potential lead compounds rather than confirmed therapeutic agents. Future studies involving in vitro PTP1B inhibition assays, cell-based experiments, pharmacokinetic analyses, and periclinal studies are required to confirm the computational findings and assess the clinical relevance of these compounds. Taken together, these theoretical findings highlight A20 as a computationally promising lead candidate with a strong molecular binding affinity, conformational stabilizing capability, favorable electronic configuration, and drug-likeness property, thereby underscoring its relevance as a potential candidate for therapeutic development against AD therapy.

## Supplementary Information

Below is the link to the electronic supplementary material.

Supplementary Material 1

## Data Availability

All generated and analysed data in this research is available upon demand to the corresponding author.

## Abbreviations

AD: Alzheimer’s disease
PTP1B: Protein tyrosine phosphatase-1β
RMSD: Root mean square deviation
RMSF: Root mean square fluctuation
H-bond: Hydrogen bond
Rg: Radius of gyration
SASA: Solvent accessible surface area
PCA: Principal component analysis
MM-PBSA: Molecular mechanics/poisson–boltzmann surface area
DFT: Density functional theory
FDA: Food and drug administration
NMDAR: N-methyl-D-aspartate
Aβ: Amyloid-beta
PTPN1: Protein tyrosine phosphatase non-receptor type 1
PTP: Protein tyrosine phosphatase
TrkB: Tropomyosin receptor kinase B
BDNF: Brain-derived neurotropic factor
SDs: Simufilam derivatives
BP: Biological process
MF: Molecular function
CC: Cellular component
GO: Gene ontology
MD: Molecular docking
MD: Molecular dynamics
HOMO: Highest occupied molecular orbital
LUMO: Lowest unoccupied molecular orbital
NBO: Natural bond orbital

## References

[CR1] Abraham MJ, Murtola T, Schulz R et al (2015) GROMACS: high performance molecular simulations through multi-level parallelism from laptops to supercomputers. SoftwareX 1:19–25. 10.1016/j.softx.2015.06.001

[CR2] Abu-Izneid T, Rauf A, Ahmad Z et al (2024) Density functional theory (DFT), molecular docking, and xanthine oxidase inhibitory studies of dinaphthodiospyrol S from *Diospyros kaki* L. Saudi Pharm J 32:101936. 10.1016/j.jsps.2023.101936 PMC10797153 38261938

[CR3] Abubakar MD, Alom S, Ali F et al (2026) Molecular Mechanisms of Dopaminergic Neuron Degeneration in Parkinson’s disease: A Comprehensive Review. Curr Neuropharmacol. 10.2174/011570159X446831260303200516 PMC13647700 41968682

[CR4] Agrawal M, Saraf S, Saraf S et al (2018) Nose-to-brain drug delivery: An update on clinical challenges and progress towards approval of anti-Alzheimer drugs. J Controlled Release 281:139–177. 10.1016/j.jconrel.2018.05.011 29772289

[CR5] Akhtar A, Sah SP (2020) Insulin signaling pathway and related molecules: role in neurodegeneration and Alzheimer’s disease. Neurochem Int 135:104707. 10.1016/j.neuint.2020.104707 32092326

[CR6] Al-Adilee KJ, Jawad SH, Kyhoiesh HAK, Hassan HM (2024) Synthesis, characterization, biological applications, and molecular docking studies of some transition metal complexes with azo dye ligand derived from 5-methyl imidazole. J Mol Struct 1295:136695. 10.1016/j.molstruc.2023.136695

[CR7] Ali R, Gupta GD, Chawla PA (2022) Aducanumab: a new hope in Alzheimer’s disease. Health Sci Rev 4:100039. 10.1016/j.hsr.2022.100039

[CR8] Aslam Mohd, Malakar VK, Nand B et al (2025) A comparative in silico study of noscapine, bosutinib, and the co-crystal ligand (DHHE) of 3B99 to investigate their interaction with prostacyclin synthase. Chem Pap. 10.1007/s11696-025-04126-0

[CR9] Baroroh SSi, MBiotek U, Muscifa ZS, Destiarani W et al (2023) Molecular interaction analysis and visualization of protein-ligand docking using Biovia Discovery Studio Visualizer. Indonesian J Comput Biology (IJCB) 2:22. 10.24198/ijcb.v2i1.46322

[CR200] Birks J, Harvey RJ (2006) Donepezil for dementia due to Alzheimer’s disease. Cochrane Database Syst Rev 10.1002/14651858.CD001190.pub2 16437430

[CR10] Bjelkmar P, Larsson P, Cuendet MA et al (2010) Implementation of the CHARMM force field in GROMACS: analysis of protein stability effects from correction maps, virtual interaction sites, and water models. J Chem Theory Comput 6:459–466. 10.1021/ct900549r 26617301

[CR11] Brandão TAS, Johnson SJ, Hengge AC (2012) The molecular details of WPD-loop movement differ in the protein-tyrosine phosphatases YopH and PTP1B. Arch Biochem Biophys 525:53–59. 10.1016/j.abb.2012.06.002 PMC3422214 22698963

[CR12] Brockmann R, Nixon J, Love BL, Yunusa I (2023) Impacts of FDA approval and Medicare restriction on antiamyloid therapies for Alzheimer’s disease: patient outcomes, healthcare costs, and drug development. Lancet Reg Health - Americas 20:100467. 10.1016/j.lana.2023.100467 PMC9996432 36908502

[CR13] Carbone G, Bencivenga L, Santoro MA et al (2024) Impact of serum leptin and adiponectin levels on brain infarcts in patients with mild cognitive impairment and Alzheimer’s disease: a longitudinal analysis. Front Endocrinol (Lausanne). 10.3389/fendo.2024.1389014 PMC11056582 38686200

[CR14] Carreira EM, Fessard TC (2014) Four-Membered Ring-Containing Spirocycles: Synthetic Strategies and Opportunities. Chem Rev 114:8257–8322. 10.1021/cr500127b 25003801

[CR15] Cen Y, Alves SR, Song D et al (2025) PTP1B inhibition promotes microglial phagocytosis in Alzheimer’s disease models by enhancing SYK signaling 10.1073/pnas.2521944123 PMC12875655 41628337

[CR16] Chen RM, Hu LH, An TY et al (2002) Natural PTP1B Inhibitors from broussonetia papyrifera. Bioorg Med Chem Lett 12:3387–3390. 10.1016/S0960-894X(02)00757-6 12419367

[CR17] Choy MS, Li Y, Machado LESF et al (2017a) Conformational Rigidity and Protein Dynamics at Distinct Timescales Regulate PTP1B Activity and Allostery. Mol Cell 65:644–658e5. 10.1016/j.molcel.2017.01.014 PMC5325675 28212750

[CR18] Combs CK, Karlo JC, Kao S-C, Landreth GE (2001) β-Amyloid Stimulation of Microglia and Monocytes Results in TNFα-Dependent Expression of Inducible Nitric Oxide Synthase and Neuronal Apoptosis. J Neurosci 21:1179–1188. 10.1523/JNEUROSCI.21-04-01179.2001 PMC6762255 11160388

[CR19] Congdon EE, Ji C, Tetlow AM et al (2023) Tau-targeting therapies for Alzheimer disease: current status and future directions. Nat Rev Neurol 19:715–736. 10.1038/s41582-023-00883-2 PMC10965012 37875627

[CR202] Cummings J, Zhou Y, Lee G, et al (2023) Alzheimer’s disease drug development pipeline: 2023. Alzheimer’s & Dementia: Translational Research & Clinical Interventions 9. 10.1002/trc2.12385 PMC10210334 37251912

[CR201] van Dyck CH, Swanson CJ, Aisen P, et al (2023) Lecanemab in Early Alzheimer’s Disease. N Engl J Med 388:9–21. 10.1056/NEJMoa2212948 36449413

[CR20] Eberhardt J, Santos-Martins D, Tillack AF, Forli S (2021) AutoDock Vina 1.2.0: New Docking Methods, Expanded Force Field, and Python Bindings. J Chem Inf Model 61:3891–3898. 10.1021/acs.jcim.1c00203 PMC10683950 34278794

[CR21] Egan MF, Kost J, Voss T et al (2019) Randomized Trial of Verubecestat for Prodromal Alzheimer’s Disease. N Engl J Med 380:1408–1420. 10.1056/NEJMoa1812840 PMC6776078 30970186

[CR22] Esmaili E, Shahlaei M (2015) Analysis of the flexibility and stability of the structure of magainin in a bilayer, and in aqueous and nonaqueous solutions using molecular dynamics simulations. J Mol Model 21:73. 10.1007/s00894-015-2622-4 25750019

[CR23] Fan F, Liu H, Shi X et al (2022) The Efficacy and Safety of Alzheimer’s Disease Therapies: An Updated Umbrella Review. J Alzheimer’s Disease 85:1195–1204. 10.3233/JAD-215423 34924395

[CR24] Friedman AJ, Liechty ET, Kramer L et al (2022) Allosteric Inhibition of PTP1B by a Nonpolar Terpenoid. J Phys Chem B 126:8427–8438. 10.1021/acs.jpcb.2c05423 PMC10040085 36223525

[CR25] Gao Y, Liu Y, Li Y (2024) Safety and efficacy of acetylcholinesterase inhibitors for Alzheimer’s disease: A systematic review and meta-analysis. Adv Clin Experimental Med 33:1179–1187. 10.17219/acem/176051 38439609

[CR26] Ghahremanian S, Rashidi MM, Raeisi K, Toghraie D (2022) Molecular dynamics simulation approach for discovering potential inhibitors against SARS-CoV-2: a structural review. J Mol Liq 354:118901. 10.1016/j.molliq.2022.118901 PMC8916543 35309259

[CR27] Gomes S, Martins I, Fonseca ACRG et al (2014) Protective Effect of Leptin and Ghrelin against Toxicity Induced by Amyloid-β Oligomers in a Hypothalamic cell Line. J Neuroendocrinol 26:176–185. 10.1111/jne.12138 24528254

[CR28] Grijaldo SJB, Alvarez MRS, Heralde FM et al (2023) Integrating computational methods in network pharmacology and in silico screening to uncover multi-targeting phytochemicals against aberrant protein glycosylation in lung cancer. ACS Omega 8:20303–20312. 10.1021/acsomega.2c07542 PMC10268607 37332828

[CR29] Hampel H, Vassar R, De Strooper B et al (2021) The β-secretase BACE1 in Alzheimer’s disease. Biol Psychiatry 89:745–756. 10.1016/j.biopsych.2020.02.001 PMC7533042 32223911

[CR30] Huq AKMM, Roney M, Dubey A et al (2024) Phenolic compounds of *Theobroma cacao* L. show potential against dengue RdRp protease enzyme inhibition by In-silico docking, DFT study, MD simulation and MMGBSA calculation. PLoS One 19:e0299238. 10.1371/journal.pone.0299238 PMC10939188 38483871

[CR31] Ishak S, Aris S, Halim K et al (2017) Molecular dynamic simulation of space and earth-grown crystal structures of thermostable T1 lipase *Geobacillus zalihae* revealed a better structure. Molecules 22:1574. 10.3390/molecules22101574 PMC6151610 28946656

[CR205] Jain V, Bharti S (2025) Mechanistic Insights into Long-Chain PFAS-Induced Toxicity: An In Silico Study Using Network Pharmacology, Molecular Docking, and Molecular Dynamics. J Indian Chem Soc 102261. 10.1016/j.jics.2025.102261

[CR32] Jin T, Yu H, Huang X-F (2016) Selective binding modes and allosteric inhibitory effects of lupane triterpenes on protein tyrosine phosphatase 1B. Sci Rep 6:20766. 10.1038/srep20766 PMC4749975 26865097

[CR33] Jindal A, Rai SN (2026) Targeting autophagy, ferroptosis, and neuroinflammation: polyphenol-mediated modulation of emerging signaling pathways in neurodegeneration. Mol Neurobiol. 10.1007/s12035-026-05760-9 41746549

[CR34] Kaushik M, Potdar M, Madeswaraguptha P et al (2026a) Mitochondrial function in neurons and glia in health and its alteration in Parkinson’s disease: a review. Curr Neuropharmacol. 10.2174/011570159X415585260303184042 PMC13536663 42163657

[CR35] Kaushik M, Vanangamudi M, Sharma S et al (2026b) From nutrients to health outcomes: integrating diet, disease risk, and lifelong wellness. 3 Biotech 16:206. 10.1007/s13205-026-04837-5 PMC13190980 42179921

[CR36] Kharwar A, Velázquez-Martínez CA, Tiwari AK (2025) Machine learning-assisted *in silico* discovery of PDE10A inhibitors: integration of QSAR modeling, docking and MD simulations. J Indian Chem Soc 102:102008. 10.1016/j.jics.2025.102008

[CR204] Knopman DS, Jones DT, Greicius MD (2021) Failure to demonstrate efficacy of aducanumab: An analysis of the EMERGE and ENGAGE trials as reported by Biogen, December 2019. Alzheimer’s & Dementia 17:696–701. 10.1002/alz.12213 33135381

[CR37] Krasemann S, Madore C, Cialic R et al (2017) The TREM2-APOE pathway drives the transcriptional phenotype of dysfunctional microglia in neurodegenerative diseases. Immunity 47:566-581.e9. 10.1016/j.immuni.2017.08.008 PMC5719893 28930663

[CR38] Krishnan N, Koveal D, Miller DH et al (2014) Targeting the disordered C terminus of PTP1B with an allosteric inhibitor. Nat Chem Biol 10:558–566. 10.1038/nchembio.1528 PMC4062594 24845231

[CR39] Kumar GS, Page R, Peti W (2020) The mode of action of the protein tyrosine phosphatase 1B inhibitor Ertiprotafib. PLoS One 15:e0240044. 10.1371/journal.pone.0240044 PMC7531832 33007022

[CR40] Kumari A, Mittal L, Srivastava M et al (2021) Conformational characterization of the co-activator binding site revealed the mechanism to achieve the bioactive state of FXR. Front Mol Biosci. 10.3389/fmolb.2021.658312 PMC8439381 34532338

[CR41] Lauretti E, Dincer O, Praticò D (2020) Glycogen synthase kinase-3 signaling in Alzheimer’s disease. Biochimica et Biophysica Acta (BBA) - Molecular Cell Research 1867:118664. 10.1016/j.bbamcr.2020.118664 PMC7047718 32006534

[CR42] Law RJ, Capener C, Baaden M et al (2005) Membrane protein structure quality in molecular dynamics simulation. J Mol Graph Model 24:157–165. 10.1016/j.jmgm.2005.05.006 16102990

[CR43] Lemkul JA (2024) Introductory Tutorials for Simulating Protein Dynamics with GROMACS. J Phys Chem B 128:9418–9435. 10.1021/acs.jpcb.4c04901 PMC11457149 39305267

[CR44] Liu S-L, Wang C, Jiang T et al (2016) The Role of Cdk5 in Alzheimer’s Disease. Mol Neurobiol 53:4328–4342. 10.1007/s12035-015-9369-x 26227906

[CR45] Liu P-P, Xie Y, Meng X-Y, Kang J-S (2019) History and progress of hypotheses and clinical trials for Alzheimer’s disease. Signal Transduct Target Ther 4:29. 10.1038/s41392-019-0063-8 PMC6799833 31637009

[CR46] Liu R, Mathieu C, Berthelet J et al (2022) Human protein tyrosine phosphatase 1B (PTP1B): from structure to clinical inhibitor perspectives. Int J Mol Sci 23:7027. 10.3390/ijms23137027 PMC9266911 35806030

[CR47] Lobanov MI, Bogatyreva NS, Galzitskaia OV (2008) [Radius of gyration is indicator of compactness of protein structure]. Molekuliarnaia biologiia 42:701–706 18856071

[CR48] Lu S, Nasrallah HA (2018) The use of memantine in neuropsychiatric disorders: An overview. Ann Clin Psychiatry 30:234–248 30028898

[CR49] Makarewicz T, Kaźmierkiewicz R (2013) Molecular Dynamics Simulation by GROMACS Using GUI Plugin for PyMOL. J Chem Inf Model 53:1229–1234. 10.1021/ci400071x 23611462

[CR50] Malakar V, Dhanabal S, Gowramma B et al (2025a) Deciphering potent protein tyrosine phosphatase-1B inhibitors through in silico molecular docking, MMGBSA, and molecular dynamics. ChemistrySelect. 10.1002/slct.202403082

[CR51] Malakar V, Dhanabal S, Justin A et al (2025b) Exploring the multi-target effects of *Valeriana officinalis* in Alzheimer’s disease: a network pharmacology approach. ChemistrySelect. 10.1002/slct.202403078

[CR52] Malakar VK, Roy D, Malakar CC et al (2025c) Targeting IDO1 in Huntington’s disease: network pharmacology and preclinical evidence from *Coffea arabica*. Neurochem Res 50:263. 10.1007/s11064-025-04509-5 40801953

[CR53] Malakar V, Dhanabal SP, Roy D et al (2026a) Network pharmacology and computational-based approaches to activate NRF2 pathway via KEAP1 and GSK-3β inhibition: Exploring the possible molecular insights of mangiferin for Alzheimer’s. Comput Biol Med 204:111507. 10.1016/j.compbiomed.2026.111507 41637820

[CR54] Malakar V, Malakar CC, Mali PC, Poddar NK (2026b) Targeting protein–protein interactions in the BCL-2 family: opportunities for precision oncology. Clin Transl Oncol. 10.1007/s12094-026-04526-y 42570165

[CR55] Malakar V, Roy D, Dugar N et al (2026c) Computational investigation of biochanin a targeting DEPTOR in Alzheimer’s disease with in vitro cellular validation of neuroprotective activity. Sci Rep. 10.1038/s41598-026-47343-7 PMC13230573 41963454

[CR56] Malakar V, Sankhla B, Malakar CC et al (2026d) Withanolides as molecular inhibitors in oral squamous cell carcinoma: unveiling anticancer mechanisms and therapeutic targets. Phytochem Rev. 10.1007/s11101-026-10240-y

[CR57] Martínez L (2015) Automatic identification of mobile and rigid substructures in molecular dynamics simulations and fractional structural fluctuation analysis. PLoS One 10:e0119264. 10.1371/journal.pone.0119264 PMC4376797 25816325

[CR58] Meanwell NA (2011) Synopsis of Some Recent Tactical Application of Bioisosteres in Drug Design. J Med Chem 54:2529–2591. 10.1021/jm1013693 21413808

[CR59] Medimagh M, Mleh CB, Issaoui N et al (2023) DFT and molecular docking study of the effect of a green solvent (water and DMSO) on the structure, MEP, and FMOs of the 1-ethylpiperazine-1,4-diium bis(hydrogenoxalate) compound. J Mol Liq 369:120851. 10.1016/j.molliq.2022.120851

[CR60] Morris GM, Huey R, Lindstrom W et al (2009) AutoDock4 and AutoDockTools4: Automated docking with selective receptor flexibility. J Comput Chem 30:2785–2791. 10.1002/jcc.21256 PMC2760638 19399780

[CR61] Nand Rai S (2026) Vitamins and Neurodegeneration. CRC Press, New York

[CR62] Ni B, Wang H, Khalaf HKS et al (2024) AutoDock-SS: AutoDock for Multiconformational Ligand-Based Virtual Screening. J Chem Inf Model 64:3779–3789. 10.1021/acs.jcim.4c00136 PMC11094722 38624083

[CR63] Olivares D, Deshpande VK, Shi Y et al (2012) N-Methyl D-Aspartate (NMDA) receptor antagonists and memantine treatment for Alzheimer’s disease, vascular dementia and Parkinson’s disease. Curr Alzheimer Res 9:746–758. 10.2174/156720512801322564 PMC5002349 21875407

[CR64] Ortiz-Islas E, Montes P, Rodríguez-Pérez CE et al (2025) Evolution of Alzheimer’s disease therapeutics: from conventional drugs to medicinal plants, immunotherapy, microbiotherapy and nanotherapy. Pharmaceutics 17:128. 10.3390/pharmaceutics17010128 PMC11768419 39861773

[CR65] Ozek C, Kanoski SE, Zhang Z-Y et al (2014) Protein-tyrosine phosphatase 1B (PTP1B) is a novel regulator of central brain-derived neurotrophic factor and tropomyosin receptor kinase B (TrkB) signaling. J Biol Chem 289:31682–31692. 10.1074/jbc.M114.603621 PMC4231649 25288805

[CR66] Pajouhesh H, Lenz GR (2005) Medicinal chemical properties of successful central nervous system drugs. NeuroRX 2:541–553. 10.1602/neurorx.2.4.541 PMC1201314 16489364

[CR67] Patel S, Prajapati C, Rai SN, Singh SK (2026) Bridging pathway dysfunction and therapy: novel compounds for neuroprotection. 3 Biotech 16:79. 10.1007/s13205-026-04697-z PMC12819964 41574214

[CR68] Pei J-J, Sersen E, Iqbal K, Grundke-Iqbal I (1994) Expression of protein phosphatases (PP-1, PP-2A, PP-2B and PTP-1B) and protein kinases (MAP kinase and P34cdc2) in the hippocampus of patients with Alzheimer disease and normal aged individuals. Brain Res 655:70–76. 10.1016/0006-8993(94)91598-9 7812792

[CR69] Qin Z, Pandey NR, Zhou X et al (2015) Functional properties of Claramine: A novel PTP1B inhibitor and insulin-mimetic compound. Biochem Biophys Res Commun 458:21–27. 10.1016/j.bbrc.2015.01.040 25623533

[CR70] Quayum ST, Esha NJI, Siraji S et al (2024) Exploring the effectiveness of flavone derivatives for treating liver diseases: utilizing DFT, molecular docking, and molecular dynamics techniques. MethodsX 12:102537. 10.1016/j.mex.2023.102537 PMC10828815 38299040

[CR71] Rahman SO, Panda BP, Parvez S et al (2019) Neuroprotective role of astaxanthin in hippocampal insulin resistance induced by Aβ peptides in animal model of Alzheimer’s disease. Biomed Pharmacother 110:47–58. 10.1016/j.biopha.2018.11.043 30463045

[CR72] Raksha C, Ansiya N, Sivan A (2025) Integrated DFT, topological, and ADME analysis of isatin-based Schiff bases: synthesis and in silico antidiabetic evaluation. J Indian Chem Soc 102:102105. 10.1016/j.jics.2025.102105

[CR73] Reddy PH, Swerdlow RH, Culberson J et al (2019) Current Status of Healthy Aging and Dementia Research: A Symposium Summary. J Alzheimer’s Disease 72:S11–S35. 10.3233/JAD-190252 31104030

[CR74] Roy A, Anbarasu A (2025) Unveiling berberine analogues as potential inhibitors of *Escherichia coli* FtsZ through machine learning molecular docking and molecular dynamics approach. Sci Rep 15:14668. 10.1038/s41598-025-98835-x PMC12033256 40287515

[CR75] Saeed M, Shoaib A, Tasleem M et al (2024) Role of Alkannin in the Therapeutic Targeting of Protein-Tyrosine Phosphatase 1B and Aldose Reductase in Type 2 Diabetes: An In Silico and In Vitro Evaluation. ACS Omega. 10.1021/acsomega.4c00082 PMC11359625 39220541

[CR76] Sarkar A, Concilio S, Sessa L et al (2024) Advancements and novel approaches in modified AutoDock Vina algorithms for enhanced molecular docking. Results Chem 7:101319. 10.1016/j.rechem.2024.101319

[CR77] Schake P, Bolz SN, Linnemann K, Schroeder M (2025) PLIP 2025: introducing protein–protein interactions to the protein–ligand interaction profiler. Nucleic Acids Res 53:W463–W465. 10.1093/nar/gkaf361 PMC12230730 40347107

[CR78] Sharallah OA, Malakar V, Salama AE et al (2026) Narrative Review Dual Frontiers in Neurodegeneration: Pharmacological Synergies and Divergences in Parkinson’s Disease and Type 2 Diabetes Mellitus. Cell Mol Neurobiol 46:86. 10.1007/s10571-026-01702-0 PMC13172232 41931123

[CR79] Shekarkhand M, Zare K, Monajjemi M et al (2022) Computational study of heterocyclic anticancer compounds through nbo method. Nexo Revista Científica 35:367–381. 10.5377/nexo.v35i01.13982

[CR203] Sims JR, Zimmer JA, Evans CD et al (2023) Donanemab in Early Symptomatic Alzheimer Disease. JAMA 330:512. 10.1001/jama.2023.13239 PMC10352931 37459141

[CR80] Singh S, Singh Grewal A, Grover R et al (2022) Recent updates on development of protein-tyrosine phosphatase 1B inhibitors for treatment of diabetes, obesity and related disorders. Bioorg Chem 121:105626. 10.1016/j.bioorg.2022.105626 35255350

[CR81] Singh B, Day CM, Abdella S, Garg S (2024) Alzheimer’s disease current therapies, novel drug delivery systems and future directions for better disease management. J Controlled Release 367:402–424. 10.1016/j.jconrel.2024.01.047 38286338

[CR82] Song GJ, Jung M, Kim J-H et al (2016) A novel role for protein tyrosine phosphatase 1B as a positive regulator of neuroinflammation. J Neuroinflammation 13:86. 10.1186/s12974-016-0545-3 PMC4837589 27095436

[CR83] Srivastava A, Renna HA, Hossain T et al (2026) Simufilam in Alzheimer’s disease: assessment of efficacy of a controversial drug in human neuronal cell culture. Pharmaceuticals (Basel) 19:281. 10.3390/ph19020281 PMC12944517 41754821

[CR84] Syed YY (2020) Sodium Oligomannate: First Approval. Drugs 80:441–444. 10.1007/s40265-020-01268-1 32020555

[CR85] Thornton S, Crane T (2020) Laboratory confirmed massive donepezil ingestion. Toxicology Cases for the Clinical and Forensic Laboratory. Elsevier, pp 485–487

[CR86] Tonks NK (2006) Protein tyrosine phosphatases: From genes, to function, to disease. Nat Rev Mol Cell Biol 7:833–846 10.1038/nrm2039 17057753

[CR87] Valdés-Tresanco MS, Valdés-Tresanco ME, Valiente PA, Moreno E (2020) AMDock: a versatile graphical tool for assisting molecular docking with Autodock Vina and Autodock4. Biol Direct 15:12. 10.1186/s13062-020-00267-2 PMC7493944 32938494

[CR88] Vijayakumar BG, Ramesh D, Joji A et al (2020) In silico pharmacokinetic and molecular docking studies of natural flavonoids and synthetic indole chalcones against essential proteins of SARS-CoV-2. Eur J Pharmacol 886:173448. 10.1016/j.ejphar.2020.173448 PMC7406432 32768503

[CR89] Wager TT, Hou X, Verhoest PR, Villalobos A (2010) Moving beyond Rules: The Development of a Central Nervous System Multiparameter Optimization (CNS MPO) Approach To Enable Alignment of Druglike Properties. ACS Chem Neurosci 1:435–449. 10.1021/cn100008c PMC3368654 22778837

[CR90] Wang H-Y, Bakshi K, Frankfurt M et al (2012) Reducing Amyloid-Related Alzheimer’s Disease Pathogenesis by a Small Molecule Targeting Filamin A. J Neurosci 32:9773–9784. 10.1523/JNEUROSCI.0354-12.2012 PMC6621293 22815492

[CR91] Wang H-Y, Lee K-C, Pei Z et al (2017) PTI-125 binds and reverses an altered conformation of filamin A to reduce Alzheimer’s disease pathogenesis. Neurobiol Aging 55:99–114. 10.1016/j.neurobiolaging.2017.03.016 28438486

[CR92] Wang X, Sun G, Feng T et al (2019) Sodium oligomannate therapeutically remodels gut microbiota and suppresses gut bacterial amino acids-shaped neuroinflammation to inhibit Alzheimer’s disease progression. Cell Res 29:787–803. 10.1038/s41422-019-0216-x PMC6796854 31488882

[CR93] Wang H-Y, Pei Z, Lee K-C et al (2020) PTI-125 Reduces Biomarkers of Alzheimer’s Disease in Patients. J Prev Alzheimers Dis 7:256–264. 10.14283/jpad.2020.6 32920628

[CR94] Wang H-Y, Cecon E, Dam J et al (2023a) Simufilam Reverses Aberrant Receptor Interactions of Filamin A in Alzheimer’s Disease. Int J Mol Sci 24:13927. 10.3390/ijms241813927 PMC10531384 37762230

[CR95] Wang H-Y, Pei Z, Lee K-C et al (2023bb) Simufilam suppresses overactive mTOR and restores its sensitivity to insulin in Alzheimer’s disease patient lymphocytes. Front Aging. 10.3389/fragi.2023.1175601 PMC10339288 37457922

[CR96] Wiesmann C, Barr KJ, Kung J et al (2004) Allosteric inhibition of protein tyrosine phosphatase 1B. Nat Struct Mol Biol 11:730–737. 10.1038/nsmb803 15258570

[CR97] Zhang N, Gordon ML (2018) Clinical efficacy and safety of donepezil in the treatment of Alzheimer’s disease in Chinese patients. Clin Interv Aging 13:1963–1970. 10.2147/CIA.S159920 PMC6187917 30349215

[CR98] Zhang J, Shen Q, Lu J-C et al (2010) Phenolic compounds from the leaves of Cyclocarya paliurus (Batal.) Ijinskaja and their inhibitory activity against PTP1B. Food Chem 119:1491–1496. 10.1016/j.foodchem.2009.09.031

[CR99] Zhang X, Jiang H, Li W et al (2017) Computational insight into protein tyrosine phosphatase 1B inhibition: a case study of the combined ligand- and structure-based approach. Comput Math Methods Med 2017:1–13. 10.1155/2017/4245613 PMC5758944 29441120

[CR100] Zhao X, Xiong L, She L et al (2022) The role and therapeutic implication of protein tyrosine phosphatases in Alzheimer’s disease. Biomed Pharmacother 151:113188. 10.1016/j.biopha.2022.113188 35676788

