# Supplementary material for: Network pharmacology–driven identification of PTP1β and rational design of simufilam-based allosteric inhibitors for neuroprotection in Alzheimer’s disease
Source: 3 Biotech. 2026 Oct 15;16(11):467. doi: 10.1007/s13205-026-05086-2 (PMC13672277; doi:10.1007/s13205-026-05086-2)
Supplement: Supplementary file 1 [file 13205_2026_5086_MOESM1_ESM.docx]

**Supplementary File for Publication**

**Table S1.** Simufilam drug design structures and their derivatives from A1 to A52.

|   **Basic Moieties of Simufilam** | | | | | |
| --- | --- | --- | --- | --- | --- |
| **S.No.** | **R_1_** | **R_2_** | **S.No.** | **R_1_** | **R_2_** |
| A1 | -H | -H | A27 |  | -C_2_H_5_O |
| A2 | -C_2_H_5_ | -H | A28 |  | -C_2_H_5_O |
| A3 | -H | -CH_3_ | A29 |  | -C_2_H_5_O |
| A4 | -H | -C_2_H_5_ | A30 |  | -C_2_H_5_F |
| A5 | -C_2_H_4_Cl | -H | A31 |  | -H |
| A6 | -C_2_H_4_Cl | -CH_3_ | A32 |  | -C_2_H_5_ |
| A7 | -C_2_H_4_Cl | -C_2_H_5_ | A33 |  | -C_2_H_5_ |
| A8 | -C_3_H_7_Cl | -C_2_H_5_ | A34 |  | -C_2_H_5_ |
| A9 | -C_3_H_7_Cl | -CH_3_ | A35 |  | -H |
| A10 | -C_3_H_7_Cl | -C_6_H_5_ | A36 |  | -CH_3_ |
| A11 | -C_2_H_5_ | -C_6_H_5_ | A37 |  | -C_2_H_5_ |
| A12 | -CH_3_ | -C_6_H_5_ | A38 |  | -C_2_H_5_ |
| A13 | -CH_2_Cl | -C_6_H_5_ | A39 |  | -CH_3_ |
| A14 |  | -C_6_H_5_ | A40 |  | -H |
| A15 |  | -H | A41 |  | -H |
| A16 |  | -CH_3_ | A42 |  | -CH_3_ |
| A17 |  | -C_2_H_5_ | A43 |  | -C_2_H_5_ |
| A18 |  | -C_2_H_5_ | A44 |  | -C_6_H_5_ |
| A19 |  | -C_3_H_7_ | A45 |  | -C_6_H_5_ |
| A20 |  | -H | A46 |  | -C_2_H_5_ |
| A21 |  | -CH_3_ | A47 |  | -CH_3_ |
| A22 |  | -C_2_H_5_ | A48 |  | -H |
| A23 |  | -C_3_H_7_ | A49 |  | -H |
| A24 |  | -H | A50 |  | -H |
| A25 |  | -H | A51 |  | -CH_3_ |
| A26 |  | -C_2_H_4_Cl | A52 |  | -C_2_H_5_ |

**Table S2.** Binding energy of 52 SD derivatives and PTP1β (PDB ID: 1T49) Co-crystal

| **CMPD** | **B.E. (kcal/mol)** | **CMPD** | **B.E. (kcal/mol)** | **CMPD** | **B.E. (kcal/mol)** | **CMPD** | **B.E. (kcal/mol)** |
| --- | --- | --- | --- | --- | --- | --- | --- |
| 1 | -6.7 | 14 | -8 | 27 | -7.6 | 40 | -7.5 |
| 2 | -6.6 | 15 | -7.9 | 28 | -6.8 | 41 | -7.5 |
| 3 | -6.6 | 16 | -7.4 | 29 | -7.1 | 42 | -7.1 |
| 4 | -6.4 | 17 | -7.2 | 30 | -7 | 43 | -6.7 |
| 5 | -6.6 | 18 | -7.2 | 31 | -7.5 | 44 | -7.5 |
| 6 | -6.2 | 19 | -7.7 | 32 | -7.4 | 45 | -7.8 |
| 7 | -6.6 | 20 | -10.8 | 33 | -6.9 | 46 | -6.9 |
| 8 | -6.3 | 21 | -7.2 | 34 | -7.1 | 47 | -6.8 |
| 9 | -6.2 | 22 | -7.1 | 35 | -7.2 | 48 | -7.7 |
| 10 | -7.1 | 23 | -7.4 | 36 | -7.2 | 49 | -7.2 |
| 11 | -7.2 | 24 | -7.6 | 37 | -7 | 50 | -7.6 |
| 12 | -7.2 | 25 | -8 | 38 | -6.6 | 51 | -7.2 |
| 13 | -7.2 | 26 | -9.5 | 39 | -6.9 | 52 | -7 |
| 53 | Co-crystal | | -6.0 | |  | | |

**Figure S1.** 2D & 3D interactions of protein-ligand complexes. **A)** Co-crystal, **B)** A20, and **C.** A26 protein-ligand complex.

**Figure S2.** PCA analysis of **D)** Co-crystal and **E)** A26 at different time interval like 0, 200 ns, 400, 600, 800, and 1000 ns.

**Table S3.** Thermodynamic energy parameters of active binding pocket amino acids with A20.

| **Active amino acids** | **Optimization energy (Hartree)** | **Dipole moment (Debye)** | **E_HOMO_ (Hartree)** | **E_LUMO_ (Hartree)** | **E_HOMO_ - E_LUMO_ (Hartree)** | **ΔE**  **(EHOMO**  **ELUMO)**  **(eV)** |
| --- | --- | --- | --- | --- | --- | --- |
| Asp193 | -323.75 | 3.41 | -0.25 | -0.03 | -0.21 | -5.83 |
| Glu276 | -398.95 | 3.16 | -0.24 | -0.02 | -0.21 | -5.85 |
| Thr263 | -438.28 | 3.15 | -0.25 | -0.04 | -0.21 | -5.73 |
| Tyr46 | -630.01 | 1.74 | -0.23 | -0.03 | -0.19 | -5.43 |

**Figure S3.** HOMO and LUMO quantum energy of proposed reaction mechanism.

**Table S4.** Determined HOMO and LUMO energies of proposed reaction mechanism (A20)

| **Compound** | **Optimization energy (Hartree)** | **Dipole moment (Debye)** | **E_HOMO_ (Hartree)** | **E_LUMO_ (Hartree)** | **E_HOMO_-E_LUMO_ (Hartree)** | **ΔE**  **(EHOMO**  **ELUMO)**  **(eV)** |
| --- | --- | --- | --- | --- | --- | --- |
| A | -747.22 | 2.93 | -0.21 | -0.02 | -0.19 | -5.17 |
| B | -325.92 | 4.93 | -0.23 | -0.04 | -0.19 | -5.17 |
| C | -1071.88 | 6.57 | -0.23 | -0.05 | -0.18 | -4.89 |
| D | -264.58 | 5.90 | -0.24 | -0.01 | -0.23 | -6.25 |
| E | -1260.03 | 2.90 | -0.21 | -0.02 | -0.19 | -5.17 |
| F | -731.18 | 4.07 | -0.26 | -0.06 | -0.20 | -5.44 |
| G/ A20 | -1530.38 | 4.09 | -0.21 | -0.02 | -0.19 | -5.17 |

**Figure S4.** Natural bond orbital energy of compound A20 and their interacting amino acids.
